# The Combined Effect of Hg(II) Speciation, Thiol Metabolism, and Cell Physiology on Methylmercury Formation by *Geobacter sulfurreducens*

**DOI:** 10.1021/acs.est.3c00226

**Published:** 2023-04-25

**Authors:** Mareike Gutensohn, Jeffra K. Schaefer, Elena Yunda, Ulf Skyllberg, Erik Björn

**Affiliations:** † Department of Chemistry, Umeå University, SE- 90187 Umeå, Sweden; ‡ Department of Environmental Sciences, Rutgers University, 14 College Farm Road, New Brunswick, New Jersey 08901, United States; § Department of Forest Ecology and Management, Swedish University of Agricultural Sciences, SE-901 83 Umeå, Sweden

**Keywords:** mercury methylation, low molecular mass thiols, mercury speciation, anaerobe microorganisms

## Abstract

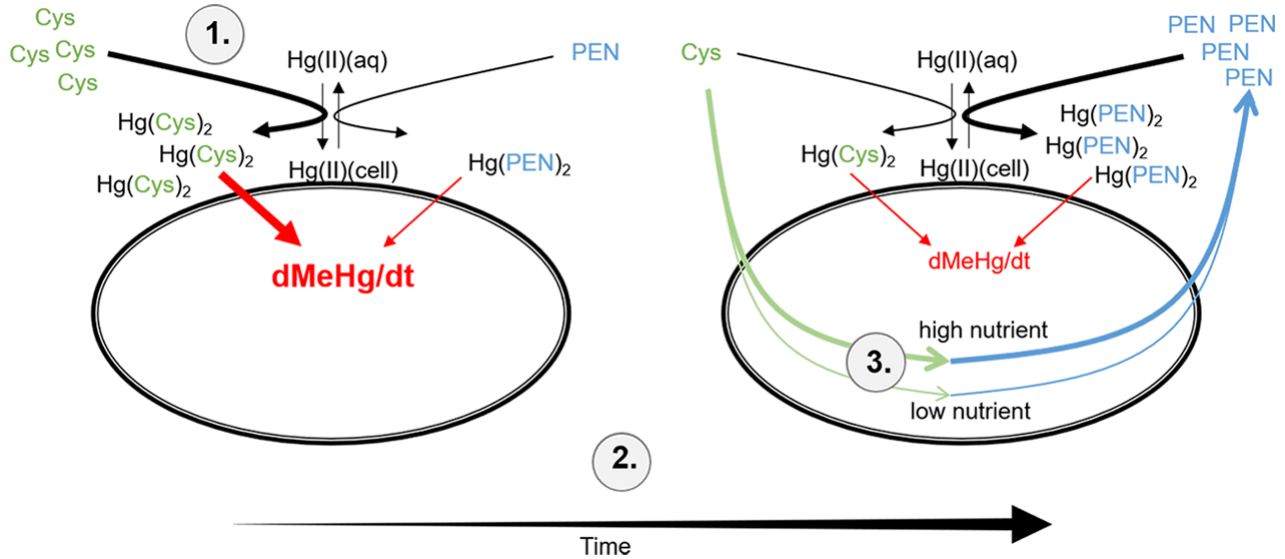

The chemical and biological factors controlling microbial formation of methylmercury (MeHg) are widely studied separately, but the combined effects of these factors are largely unknown. We examined how the chemical speciation of divalent, inorganic mercury (Hg(II)), as controlled by low-molecular-mass thiols, and cell physiology govern MeHg formation by *Geobacter sulfurreducens*. We compared MeHg formation with and without addition of exogenous cysteine (Cys) to experimental assays with varying nutrient and bacterial metabolite concentrations. Cysteine additions initially (0–2 h) enhanced MeHg formation by two mechanisms: (i) altering the Hg(II) partitioning from the cellular to the dissolved phase and/or (ii) shifting the chemical speciation of dissolved Hg(II) in favor of the Hg(Cys)_2_ complex. Nutrient additions increased MeHg formation by enhancing cell metabolism. These two effects were, however, not additive since cysteine was largely metabolized to penicillamine (PEN) over time at a rate that increased with nutrient addition. These processes shifted the speciation of dissolved Hg(II) from complexes with relatively high availability, Hg(Cys)_2_, to complexes with lower availability, Hg(PEN)_2_, for methylation. This thiol conversion by the cells thereby contributed to stalled MeHg formation after 2–6 h Hg(II) exposure. Overall, our results showed a complex influence of thiol metabolism on microbial MeHg formation and suggest that the conversion of cysteine to penicillamine may partly suppress MeHg formation in cysteine-rich environments like natural biofilms.

## Abstract 2

Interplays between chemical and biological factors controlling microbial methylmercury formation are poorly understood. This study demonstrates that metabolic turnover of thiol compounds is a key factor controlling mercury speciation and methylation.

## Introduction

The neurotoxin monomethylmercury (MeHg) is an environmental pollutant which is spread across ecosystems, bioaccumulates in the aquatic food web,^1,2^ and can lead to severe health issues.^3,4^ The major origin of MeHg in the environment is through intracellular methylation of divalent, inorganic mercury (Hg(II)) by phylogenetically diverse microorganisms.^5^ All Hg methylating microorganisms characterized so far carry the gene sequence *hgcAB,*(6,7) and a correlation between *hgcAB* expression levels and the Hg(II) methylation rate was recently demonstrated.^8^ The microbial formation of MeHg is constitutive, and the expression of *hgcAB* is not induced by Hg(II) exposure^5,9,10^ but instead controlled by a complex interplay of biological and chemical factors. These include Hg(II) speciation, availability, and uptake across the cell membrane, as well as microbial community composition and its dependence on the availability of electron donors and acceptors, temperature, pH, and redox conditions.^11^

Our understanding of the molecular mechanisms of Hg(II) uptake across the cell membrane is still incomplete despite it being a key step in the methylation of Hg(II).^12,13^ Both passive^14,15^ and active^16,17^ uptake mechanisms have been proposed. Several reports emphasize the involvement of extracellular and cell-associated thiol compounds in Hg(II) uptake and methylation.^16,18−21^ It has been consistently shown that the presence of small low-molecular-mass thiol (LMM-thiol) compounds, such as cysteine (Cys), enhance Hg(II) methylation by the two common model organisms *Geobacter sulfurreducens* and *Pseudodesulfovibrio mercurii* ND132 (ND132; formerly *Desulfovibrio desulfuricans*).^16,18,22−24^ Low molecular mass thiols with a more branched and bulky chemical structure, such as penicillamine (PEN), and mixtures of natural organic matter (NOM) have been shown to enhance Hg(II) methylation in ND132 but not in iron-reducing bacteria.^16,25,26^ Other studies have demonstrated a correlation between the concentration of LMM-thiol compounds and the Hg(II) methylation potential, or rate, in natural biofilms and wetland soils.^27−30^ The mechanisms for the observed enhancement effects and correlations are not fully clear but have been ascribed to formation of Hg(LMM-RS)_2_ complexes with high availability for cellular uptake and methylation^16,18,23,30^ and/or decreased partitioning of Hg(II) between cell-associated and dissolved Hg(II).^16,20,23,25^ Two recent studies suggest that LMM-thiol compounds affect Hg(II) methylation also by other, unidentified mechanisms, not related to the chemical speciation of dissolved Hg(II).^22,30^ Additional studies showed how nutrient concentrations and NOM composition enhance MeHg formation in the environment by increasing bacterial activity.^31−34^ In mixed consortia it was shown that MeHg formation is limited by the availability of Hg(II) under high metabolic activity, whereas under low microbial activity Hg(II) availability is not a key parameter.^35^ There are, however, few studies investigating the combined effect of Hg(II) availability and microbial activity in Hg(II) methylation experiments, despite their expected importance.

Previous studies have been conducted using experimental assay systems with relatively nutrient poor buffers,^16−23,36−40^ which may result in the physiological disturbance of bacteria cells and may influence the role of LMM-thiol compounds for MeHg formation. Further, the time-dependent changes in the concentrations of LMM-thiol compounds at varying assay conditions and the subsequent impact on time-dependent changes in Hg(II) speciation and methylation are poorly understood. Such understanding is important for accurate interpretation of experimental results investigating the roles of LMM-thiol compounds in microbial MeHg formation and to further predict MeHg formation in the environment. Especially in microenvironments like biofilms, where methylating microorganisms have been showing moderate to high methylation potential, cells are exposed to exogenous metabolites with high LMM-thiol concentrations and fluctuating nutrient availability.^27,28^

The overall aim of this study is to improve the understanding of how the combined effect of Hg(II) speciation and physiological state of bacteria cells controls MeHg formation. The first goal is to understand how Hg(II) methylation is controlled by Hg(II) speciation with LMM-thiols and by physiological states of bacteria cells in assays with varying metabolite and nutrient concentrations. The second goal is to establish if the well-documented cysteine-induced enhancement of Hg(II) methylation is consistent for contrasting assay buffer compositions and how cysteine addition affects the time-dependent speciation of dissolved Hg(II). We hypothesized that separate cysteine and nutrient additions would enhance MeHg formation by impacting Hg(II) speciation and cell physiological state, respectively, but that the two factors jointly would cause a complex response in the time-dependent MeHg formation.

## Materials and Methods

### Bacteria Culture

Geobacter sulfurreducens (ATCC 51573)^41^ was grown under a N_2_ atmosphere at 30 °C and pH 6.8 using acetate as an electron donor and fumarate as an electron acceptor in a standard growth medium as described in Schaefer and Morel (growth medium composition Table S1).^18^ Cells were grown to midexponential growth phase (OD_660_ = 0.15; *t* = 40 h), harvested by centrifugation (4000*g*; 8 min, 5 °C) in an anaerobic glove chamber under a N_2_ atmosphere (Saffron Scientific Equipment Ltd., North Yorkshire, UK), washed twice with carbon-free anoxic assay buffer and resuspended in assay buffer.^18^

### Experimental Design of Mercury Methylation Assays

Mercury methylation assays were performed in three different assay buffers in closed glass serum vials: (I) a standard assay buffer as described by Schaefer and Morel,^18^ (II) nutrient assay buffer, and (III) metabolite assay buffer (detailed assay buffer compositions are given in Table S1).^42^ The nutrient concentration increased in the order from standard < metabolite < nutrient assay with 1 mM acetate (*e*^*–*^ donor) and fumarate (*e*^*–*^ acceptor), 1.0–1.9 mM acetate and 1.0–3.9 mM fumarate, and 1.9 mM acetate and 3.9 mM fumarate for each assay system, respectively (Table S1). Assay buffers were prepared in acid-clean glass serum bottles, flushed with N_2_, crimp sealed with Teflon stoppers, and autoclaved. The pH was 6.8 in all experiments with a chloride ion concentration of 1.6 mM. The nutrient and metabolite assay buffers contained 90% (v/v) of standard assay buffer and 10% (v/v) of fresh growth medium or metabolite medium, respectively.^42^ The metabolite medium was prepared as the filtrate of a growth culture of *G. sulfurreducens* at low Fe(II) concentration (1.5 μM total Fe(II)). A sample was collected by filtration (0.2 μm pore size PES syringe filters) at the late exponential growth phase. This filtrate was added to the assay buffer (10% v/v) to comprise the metabolite medium, which contained biogenic metabolites including enhanced concentrations of cysteine and other LMM-thiols.^42^

Mercury and exogenous cysteine were added to the assay buffers to a final concentration of 30 nM Hg(II) (as HgNO_3_; Sigma-Aldrich certified standard) and 0–600 nM cysteine (anoxic solution, l-cysteine (≥97%), Sigma-Aldrich). The used Hg(II) concentration is typical for Hg(II) methylation experiments with *G. sulfurreducens*,^16,19,21−23,39,40^ and the added cysteine concentrations were chosen based on reported concentrations of this thiol in environmental samples.^22,27,28,43,44^ Demethylation of MeHg was monitored by the addition of Me^204^Hg to a final concentration of 3 nM. To selected assays, 100 nM freshly prepared sulfide (100 μM Na_2_S·9H_2_O stock in deoxygenated Milli-Q-water (>18 MΩ·cm, Merck Millipore, Sigma-Aldrich) was added. Prior to inoculation, assays were pre-equilibrated with exogenous cysteine, metabolites, and/or nutrients and Hg(II) for 1 h at the incubation temperature of 30 °C under dark conditions. Incubation assays were initiated by inoculation of 1 mL washed cell suspension (*t* = 0 h) to a final cell density of ∼10^8^ cells mL^–1^ (corresponding to an optical density of ∼0.02 at λ = 660 nm).^18^ The redox state of each assay was monitored by a pink-to-clear color change by the redox indicator resazurin at Eh ≤ −100 mV following the addition of cells. Assays that remained pink, indicating high redox potential, were not further used. All experiments were carried out in triplicate at 30 °C in the dark under anaerobic conditions, and subsamples for LMM-thiols and Hg analyses were collected at 0.5, 2, 6, and 24 h following inoculation. Samples for extracellular LMM-thiols and total dissolved Hg were collected by filtration through 0.2 μm pore size syringe filters.

### Analyses of MeHg, Hg, LMM-thiol Compounds, and Sulfide and Speciation Modeling of Hg

Quantities of MeHg and Hg were analyzed by isotope dilution analyses. For total MeHg analyses, a sample aliquot of 1 mL was collected, spiked with Me^200^Hg as an internal standard, and digested in 0.6 M NaOH for 24 h (adapted from Carrasco and Vassileva^45^). After digestion, the pH was adjusted to 4.5 with 5 M HCl and 2 M CH_3_COONH_4_ buffer (pH = 4.5). MeHg was derivatized with NaB(C_2_H_5_)_4_, purged, and trapped on Tenax adsorbent and analyzed with thermal desorption (TD-100 Thermal Desorber, Markes international) gas chromatography–inductively coupled plasma mass spectrometry (Agilent GC 7890B and Agilent 7700 ICPMS, Agilent technologies, Santa Clara, California, US).^46^

Total Hg and total dissolved Hg samples were spiked with ^200^Hg(II) (96.41%, Oak Ridge National Laboratory, TN, USA) and digested in 0.02 M BrCl for 48 h. Excess BrCl was reduced with hydroxylamine (4.3 M) followed by online SnCl_2_ reduction of Hg(II), according to EPA Method 1631E.^47^ Samples were analyzed with a CETAC HGX-200 cold vapor system (Teledyne CETAC Technologies, Omaha, Nebraska, US) coupled to an ICPMS instrument (Agilent 8900 Triple Quadrupole Inductively Coupled Plasma Mass Spectrometry, Agilent technologies, Santa Clara, California, US).

Concentrations of dissolved and cell-associated (sum of cell adsorbed and intracellular Hg) Hg(II) and MeHg were calculated from the measured total and dissolved Hg and total MeHg fractions as follows:
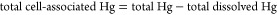
1
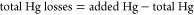
2
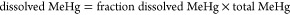
3
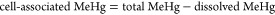
4
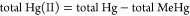
5
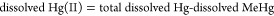
6
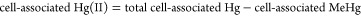
7

The “fraction dissolved MeHg” used to calculate the dissolved MeHg concentration was established based on the extracellular LMM thiol concentration at each time point and a linear approach of the experimental data by Lin et al. (see Supplemental Table S3).^23^ The quality control approaches and results are described in detail in the SI Text “Quality control and Hg recovery”.

The concentrations of specific LMM-thiol compounds in bacteria assays were reproduced from the study by Gutensohn et al.,^42^ which was partly based on samples collected from the very same experiment assays as used in this study. The LMM-thiol compounds were determined by liquid chromatography mass spectrometry following the method by Liem-Nguyen et al.^48^ Sulfide was measured in the growth culture and in specific methylation assays by the methylene blue method using UV–vis absorption spectroscopy at 670 nm (UV-1201 spectrophotometer, Shimadzu).^49,50^

The thermodynamic model developed by Liem-Nguyen et al.^51^ was adopted to calculate the chemical speciation of dissolved Hg(II) in the extracellular buffer of *G. sulfurreducens* assays at each time point using the software WinSGW.^52^ The dissolved Hg(II) speciation in *G. sulfurreducens* assays is predominantly described by complexes with LMM-thiol compounds (Hg(LMM-RS)_2_). The three dominant Hg(II) complexes are formed with cysteine, cysteamine, and penicillamine denoted as Hg(Cys)_2_, Hg(CysN)_2_ and Hg(PEN)_2_. The complete speciation data and model are given in Tables S5 and S6. We further modeled the formation of MeHg by Hg(II) species-specific, first-order rate models as described in the SI Text.

### Physiological State of *G. sulfurreducens* Assay Cultures

The cell density was monitored throughout each experiment by optical density measurements at λ = 660 nm (OD_660_) on an UV-1201 spectrophotometer (Shimadzu). The physiological state of cells was studied by monitoring changes in biochemical composition using attenuated total reflectance Fourier transform infrared (ATR-FTIR) spectroscopy by collecting 100 scans between 3996 and 698 cm^–1^ (Bruker Vertex 80v FTIR spectrometer) with a resolution of the single beam of 4 cm^–1^. At each time point (0.5, 2, 6, and 24 h) cells of standard, metabolite, and nutrient assays were collected by centrifugation (4000*g*, 16 min, 5 °C) and placed on the accessory for spectra recording. Respective supernatants were used as reference spectra. The absorbance scale of the spectra correspond to log(*R*_reference_/*R*_sample_) with *R* as the internal reflectance.

## Results and Discussion

We performed Hg(II) methylation experiments using washed *G. sulfurreducens* cells suspended in three different assay buffers with varying metabolite and nutrient concentrations: standard [1 mM acetate (*e*^*–*^ donor) and fumarate (*e*^*–*^ acceptor)], metabolite (1.0–1.9 mM acetate and 1.0–3.9 mM fumarate), and nutrient (1.9 mM acetate and 3.9 mM fumarate) assays (Table S1). In addition to the content given in Table S1, the metabolite assays also contained unknown metabolites, including enhanced concentration of cysteine and other LMM-thiols, produced and secreted by *G. sulfurreducens* during exponential growth. Under all three assay conditions, MeHg formation was studied in systems with 30 nM of Hg(II), with and without the presence of exogeneous cysteine (at concentrations of 100 and 600 nM). The complete Hg data set is given in Table S3. The concentration data for LMM-thiol compounds in Table S4 were reproduced form Gutensohn et al. and included the eight thiol compounds cysteine (Cys), cysteamine (CysN), homocysteine (HCys), mercaptoacetic acid (MAC), monothioglycerol (Glyc), penicillamine (PEN), *N*-acetyl-cysteine (NacCys), and *N*-acetyl-d-penicillamine (NacPEN).^42^ Previous studies have shown that complexes formed between Hg(II) and thiols of a smaller size and simplicity in chemical structure (e.g., cysteine) are methylated at higher rates than complexes formed with thiols having a more branched or bulky structure (e.g., penicillamine).^16,22^ We therefore grouped the thiols into “small” (Cys, CysN, HCys, MAC, Glyc) and “branched” (PEN, NacCys and NacPEN) based on their chemical structure. Since Hg(II) forms strong covalent bonds with thiol groups (RSH) but not with disulfide groups (RSSR), only the thiol form was quantified of each compound and not the corresponding organic disulfide forms (which sometimes is done by adding a reducing agent^53^). The complete reproduced LMM-thiol data set is given in Table S4.^42^

### Hg(II) Partitioning and Time-Dependent MeHg Formation

Across all assays, the recovery of added Hg(II) was 65%–85% at 0.5 h and 51%–71% at 24 h. The highest losses were observed for the unamended standard assays, while the addition of metabolites or exogenous cysteine decreased Hg-losses by >12% (Table S3). The losses were due to a combination of adsorption to glassware^54,55^ and Hg(II) reduction and volatilization of Hg(0).^19,21^ Both processes were confirmed in additional recovery experiments (see supplement “Quality control and recovery of Hg”, Figure S6). The complexation of Hg(II) with strongly bonding ligands such as LMM-thiols (added with the metabolites and exogenous cysteine) will suppress these reactions and increase Hg(II) retention in the assay, as shown in previous studies.^19,23,56^

**Figure 1:**
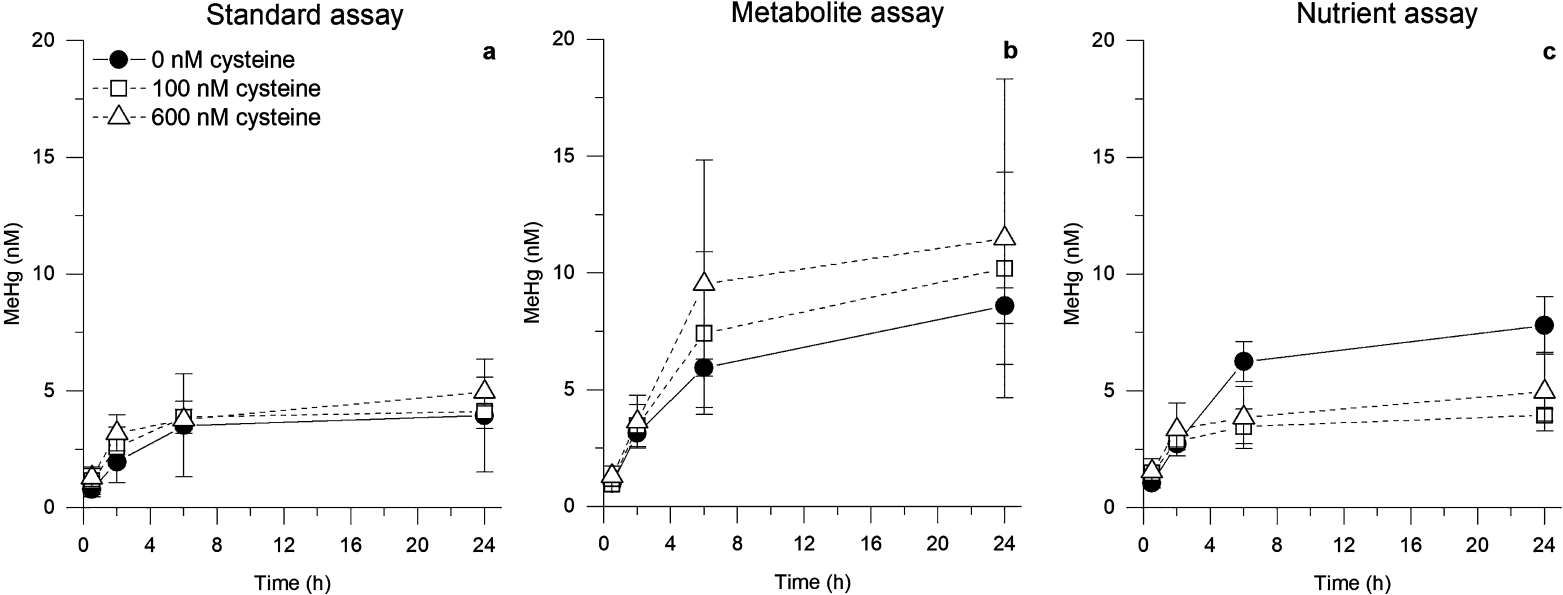
Methylmercury formation by *G. sulfurreducens* cells in the presence of 30 nM Hg(II) and addition of exogenous cysteine in the (a) standard, (b) metabolite, and (c) nutrient assay over time (*n* = 3; ± standard deviation). 0 nM cysteine (black circles), 100 nM cysteine (squares open), and 600 nM cysteine (triangles open).

**Figure 2:**
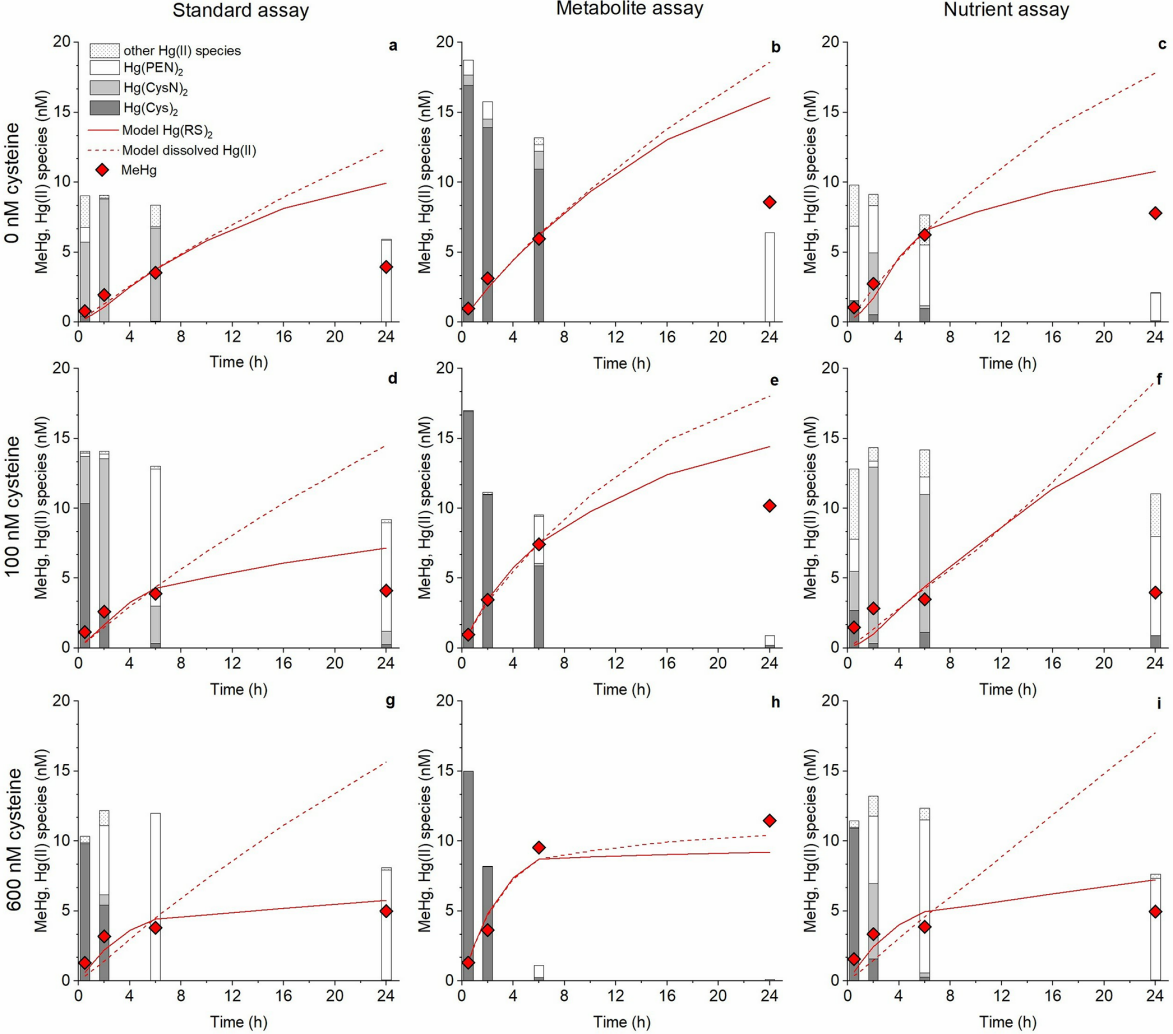
Composition of dissolved Hg(II)-species (bars) in the extracellular media of *G. sulfurreducens* in the (a, d, g) standard, (b, e, h) metabolite, and (c, f, i) nutrient assay with exogenous cysteine of 0, 100, and 600 nM after 0.5, 2, 6, and 24 h incubation time. All assay systems contained initially 30 nM of Hg(II). Dissolved Hg(II) species are Hg(Cys)_2_ (dark gray), Hg(CysN)_2_ (light gray), Hg(PEN)_2_ (white), and other Hg(II) species (white dotted). Measured concentrations of MeHg (nM) formed by *G. sulfurreducens* in each assay system over time (same data as shown in Figure 1, red diamonds). Modeled MeHg concentrations (nM) with the species-specific Hg(II) rate model (red line) and total dissolved Hg(II) rate model (dotted red line) over time based on a first-order rate model as described in the Supporting Information.

Figure 1 shows the time-dependent formation of MeHg in standard, metabolite, and nutrient assays with and without addition of exogeneous cysteine. Under all conditions, the MeHg concentration increased rapidly between 0.5 and 2 h, followed by a lower net MeHg formation at incubation times ≥2 h. The total concentration of MeHg formed during 24 h in assays without exogenous cysteine increased in the order: standard assay < nutrient assay ≈ metabolite assay (Figure 1a–c, solid line). The higher amount of MeHg produced in the metabolite compared to the standard assay (8.6 ± 0.8 vs 4.0 ± 2.4 nM, Figure 1a,b) can be explained by a higher concentration of dissolved Hg(II) complexes with small thiols in the former (Figure 2a,b), making Hg(II) more available for methylation. The metabolite medium contained ∼140–1700 nM of LMM-thiols during the incubation time (Table S4), that shifted the Hg(II) partitioning from cells to the dissolved phase (Table S3) and thus increased the concentration of available Hg(II) (Figure 2a,b, Table S5). An increased partitioning of Hg(II) from cells to the dissolved phase has previously been shown to enhance Hg(II) methylation in sulfate-reducing bacteria.^20^ A recent study with *G. sulfurreducens* showed that cell-associated Hg(II) (either sorbed to the cell surface or accumulated intracellularly) was much less available for methylation than the extracellular dissolved Hg(II) pool.^40^

The composition of Hg(II) species involving small and relatively simple-structured thiols partly differed between the standard and metabolite assays, with Hg(CysN)_2_ dominating in the former and Hg(Cys)_2_ in the latter (Table S5). Previous studies have shown that the methylation rate of these two Hg(II)-thiol complexes are very similar.^16,22^ Thus, the lower concentration of formed MeHg in the standard vs metabolite assays was more likely caused by an increase in Hg(II) partitioning toward cells in the former rather than by changes in speciation of the dissolved Hg(II) (Figure 1a,b, Figure 2a,b). These results show that changes in the distribution and concentration of Hg-binding ligands in the extracellular medium impact Hg(II) retention in the dissolved phase, an important control on Hg(II) availability and MeHg production.

The formation of MeHg in nutrient assays, however, could not be explained by higher Hg(II) availability, as more MeHg was produced in nutrient assays than standard assays (Figure 1a,c), despite having lower concentrations of more small Hg(II)-thiol complexes (Figure 2a,c). Additional experiments examining potential biological factors which could explain these results are discussed further in the section Impact of Cell Physiological State on Hg(II) Methylation among Assays.

### Time-Dependent Changes in Hg(LMM-RS)_2_ Speciation and MeHg Formation with Cysteine Addition

We investigated the effect of exogenous cysteine addition on the time-dependent MeHg formation over 24 h. In standard assays, the recovery of exogenous cysteine addition was 75–110% at 0.5 h (Table S4, Table S5). As expected, cysteine concentrations were initially higher in metabolite assays (∼90–1100 nM cysteine, Table S4), as the metabolite assay also contained endogenous thiols, in particular cysteine, produced by *G. sulfurreducens* during cell growth. This biologically produced cysteine varied substantially across batches. Lower than expected cysteine levels were detected in nutrient assays (∼10–350 nM cysteine, Table S4), suggesting rapid uptake and/or biotransformation of cysteine already within the first 0.5 h. For all treatments, the extracellular cysteine concentration rapidly declined over 24 h and penicillamine concentrations increased (Table S4). We recently demonstrated that *G. sulfurreducens* methylate cysteine to penicillamine in response to the addition of exogenous cysteine.^42^ This process has potentially substantial implications on MeHg production since it has been demonstrated that Hg(II) methylation by *G. sulfurreducens* is consistently enhanced by cysteine, but not by penicillamine.^16,22^

The response in Hg(II) methylation following the addition of 100 or 600 nM exogenous cysteine differed among the three assays. The total amount of produced MeHg was unaffected in the standard assay, increased in the metabolite assay (albeit with high variability), and decreased in the nutrient assay (Figure 1). In the standard assay, cysteine additions initially (0.5–2 h) resulted in an increased concentration of the Hg(Cys)_2_ complex (74–99% of dissolved Hg(II) present as Hg(Cys)_2,_Table S5) and thus increased availability for cellular uptake and methylation of Hg(II) (Figure 2d,g). At extended incubation times (≥2 h), however, the added cysteine was metabolized to penicillamine (Table S4) which shifted the speciation of dissolved Hg(II) from Hg(Cys)_2_ (and Hg(CysN)_2_) to Hg(PEN)_2_.^42^ This speciation shift was amplified at increased cysteine additions and decreased the availability for Hg(II) methylation (84–96% of dissolved Hg(II) present as Hg(PEN)_2_ at ≥6 h, Figure 2, Table S5). The net effect of these two opposing processes resulted in a very similar total amount of produced MeHg during 24 h at all three cysteine additions in the standard assay. In the metabolite assay, the speciation of dissolved Hg(II) was largely dominated by Hg(Cys)_2_ during the first 6 h at all three cysteine addition levels (63–99% Hg(Cys)_2_, Figure 2b,e,h, TableS5). This is explained by the presence of 100–700 nM of cysteine produced by *G. sulfurreducens* during the growth phase and added as metabolite medium, in addition to the exogeneous cysteine addition (Table S4). Formation of Hg(PEN)_2_ was observed at extended times, but at 100 and 600 nM of added exogeneous cysteine the fast methylation of Hg(II) caused a depletion in dissolved Hg(II) before a significant shift in speciation from Hg(Cys)_2_ to Hg(PEN)_2_ occurred (Figure 2b,e,h). In the nutrient assay, the additions of cysteine caused similar effects on the speciation of dissolved Hg(II) as in the standard assays. However, the metabolism of cysteine was considerably faster in the nutrient assays. Therefore, the concentration of Hg(Cys)_2_ was only enhanced at 0.5 h and 600 nM cysteine addition, as compared to the treatment with no cysteine addition (16%, 21% and 99% Hg(Cys)_2_ for 0, 100, and 600 nM cysteine addition at 0.5 h, respectively, Figure 2c,i, Table S4). The pool of Hg(II) complexes with small LMM-thiols (Figure 2) was generally dominated by complexes with cysteamine in this treatment (Figure 2c,f,i, Table S5).

To further clarify the importance of the chemical speciation shift from Hg(Cys)_2_ to Hg(PEN)_2_ to retard MeHg formation, we generated species-specific Hg(II) methylation rate models based on the time-resolved Hg(II) speciation data in Figure 2 (described in detail in SI Text). Briefly, we modeled each time interval separately (0–0.5, 0.5–2, 2–6, and 6–24 h) using first-order exponential rate models with respect to the concentration of each Hg(II)-thiol species. Notably, the values of the rate constant for the Hg(Cys)_2_ species, *k*_meth_(Hg(Cys)_2_), are comparable to rate constants for Hg(Cys)_2_ in previous studies with *G. sulfurreducens* assays (Table S2). In the standard assay the *k*_meth_(Hg(Cys)_2_) was 0.7 × 10^–12^ L cell^–1^ h^–1^ as compared to rates of 0.1–0.34 × 10^–12^ L cell^–1^ h^–1^ in previous studies (Table S2).^17,18,22^ In assays with high LMM-thiol concentrations (metabolite assay +600 nM cysteine) our fitted value was the same (1.6 × 10^–12^ L cell^–1^ h^–1^) as previously reported in methylation assays with 10 μM cysteine (Table S2).^16^ The fact that modeled rate constants are highly reproducible among studies, having quite different experimental set-ups, suggests they are mainly dependent on LMM-thiol concentrations occurring in these types of experiments, deciding the Hg(II)-speciation.

The modeling approach takes into account how MeHg formation is affected by changes in the chemical speciation of dissolved Hg(II). However, other processes occurring in parallel, that potentially affect the total concentration of dissolved Hg(II) during the assays (e.g., changes in partitioning of Hg(II) between cell and solution and losses of Hg(II)) will also affect the parametrization. To isolate the effect of the shift in chemical speciation on MeHg formation we compared the outcome of the species-specific methylation model with a model in which total dissolved Hg(II) was considered as the substrate for methylation by the bacterium.

For assays with 600 nM exogenous cysteine, the species-specific Hg(II) methylation rate model described fairly well the formation of MeHg, while the total Hg(II) methylation model largely overpredicted MeHg formation in the standard and nutrient assays (Figure 2g,i). This difference illustrates how the shift in chemical speciation from Hg(Cys)_2_ to Hg(PEN)_2_, caused by a metabolic conversion of cysteine to penicillamine, suppressed MeHg formation in these two treatments because of the 20 times lower *k*_meth_ for Hg(PEN)_2_ as compared to Hg(Cys)_2_.^16^ As noted above, this shift in Hg(II)-thiol speciation was not observed in the metabolite assay, and MeHg formation progressed therefore until the total dissolved Hg(II) was depleted (Figure 2h). This is manifested by the very similar results obtained by the species-specific and total dissolved Hg(II) rate models for the metabolite assay. In all treatments at extended time intervals (>6 h), both models overpredicted MeHg formation at lower cysteine additions, although the species-specific model still performed better than the total concentration model (Figure 2). The speciation of dissolved Hg(II) was more complex; i.e., Hg(II) was distributed among a larger number of species, in the assay buffers at lower cysteine concentrations (Figure 2, Table S5). The overpredicted MeHg formation at *t* > 6 h suggests that the speciation may have included dissolved Hg(II) complexes with unidentified ligands and with lower availability for methylation than for the Hg(Cys)_2_ complex. In case other unidentified LMM thiols are present, the thermodynamic speciation model would overestimate the concentration of Hg(Cys)_2_, and consequently both models would overpredict MeHg formation.

Unidentified ligands that could contribute to the formation of Hg(II) complexes with low availability for methylation include large thiol compounds with a “bulky” chemical structure (e.g., protein/peptide fragments), as well as inorganic sulfide. Cysteine can be microbially degraded to inorganic sulfide, and this process has been demonstrated for *G. sulfurreducens* in the presence of considerably higher cysteine concentrations (≥100 μM)^22,40^ than used in our study. Even if we did not detect sulfide (LOD 120 nM) in any of the assay conditions, nanomolar concentrations of sulfide could still be formed by degradation of cysteine or other thiols and therefore influence the speciation of the nM concentrations of Hg(II) in our assays. If sulfide mineral phases such as β-HgS(s) were formed this would decrease Hg(II) availability over time.^18,22,38,57^ In separate incubation experiments, we found that the addition of 100 nM of inorganic sulfide (below LOD) inhibited MeHg formation up to at least 6 h for all three assays (Figure S4). It can thus not be excluded that formation of sulfide over time contributed to lower than predicted MeHg formation at *t* > 6 h. The concentration of total dissolved Hg(II) did not decrease in the assays with added sulfide, suggesting that dissolved and/or nanoparticulate Hg(II)-sulfide species, HgS(s), passing the 0.2 μm filter, were formed.

In principle, MeHg demethylation could be an additional process contributing to the time trends observed in Figure 1, as commonly is the case in incubation experiments with environmental samples where a steady-state between Hg(II) methylation and MeHg demethylation is often observed at extended times.^58,59^ However, previous studies have shown that *G. sulfurreducens* does not demethylate MeHg.^23,60^ This was verified also for the three assay conditions used in our study, and we did not detect any demethylation of a Me^204^Hg tracer added to the assays (Figure S5; no significant differences between time points of different samples; repeated measure ANOVA, *p* > 0.05). Demethylation was thus not a process contributing to the observed time-dependency of MeHg formation.

To summarize, the results in Figures 1 and 2 and Tables S2–S5 demonstrate that the time-dependent effect of exogenous cysteine additions on Hg(II) methylation by *G. sulfurreducens* is governed by Hg(II) partitioning between the cellular and aqueous phases and the distribution of Hg(II) between complexes formed with small and branched thiols. These processes are in turn largely controlled by the assay-specific metabolic turnover of the added cysteine, and the results highlight the importance of monitoring the fate of added cysteine for accurate interpretation of its impact on Hg(II) methylation.

### Impact of Cell Physiological State on Hg(II) Methylation among Assays

As discussed above, the elevated MeHg formation observed at 0 nM of exogenous cysteine in the nutrient assay (Figures 1 and 2a,c) in relation to the standard assay could not be explained by Hg(II) speciation effects. We investigated the physiological state and growth of cells among the treatments to further clarify the differences in methylation.

**Figure 3:**
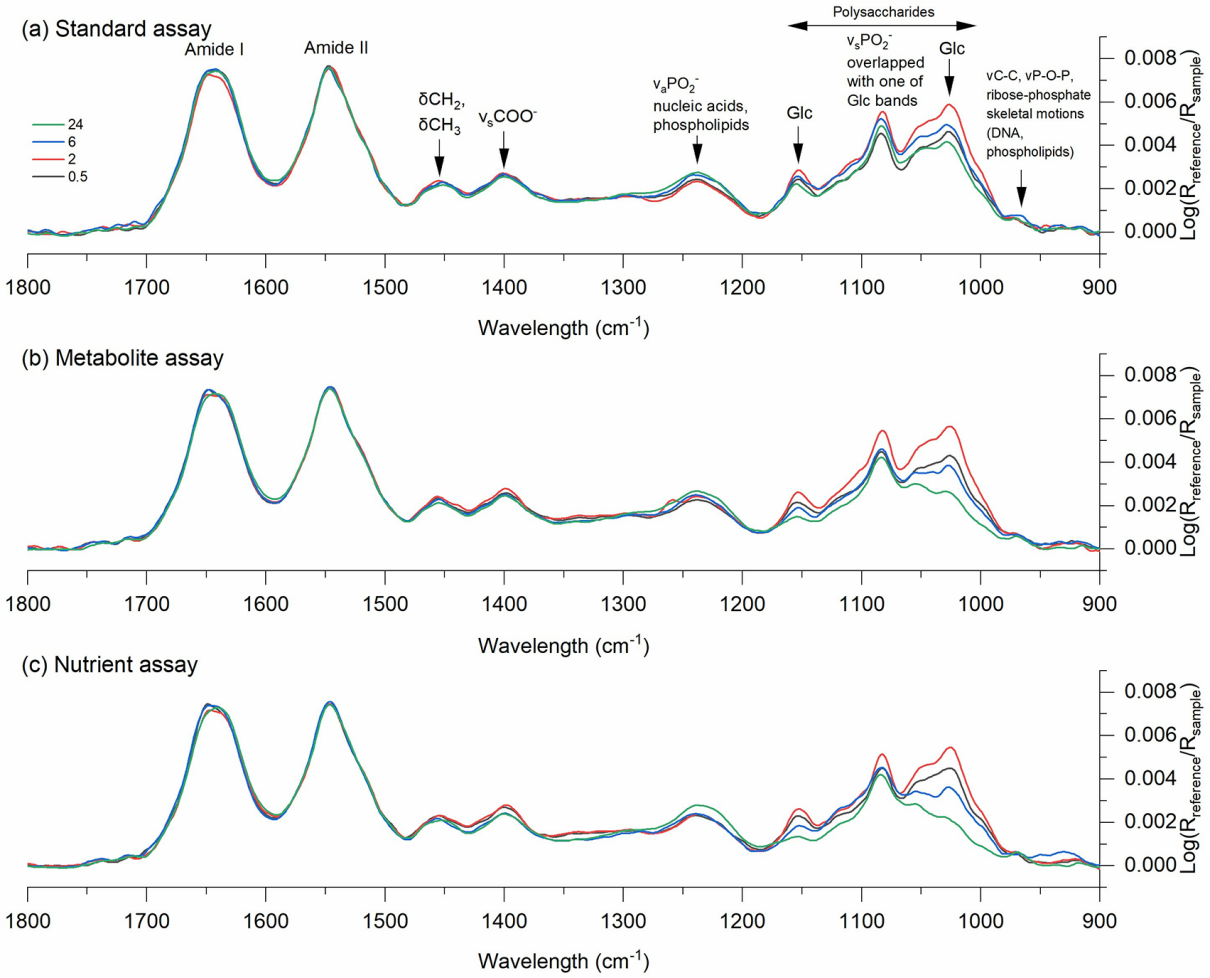
Evolution over time of ATR-FTIR spectra collected from 0.5 to 24 h bacterial suspension of *G. sulfurreducens* cells with 30 nM Hg(II) in (a) standard, (b) metabolite, and (c) nutrient assays. Spectra were recorded after 0.5, 2, 6, and 24 h incubation times and indicated in black, red, blue, and green, respectively. Corresponding supernatants at each time point were used as the reference spectra after centrifugation of the sample. The spectra were baseline corrected and normalized to the amide II band at 1548 cm^–1^. Principle assignments of the infrared bands are indicated in the spectra and based on Quilès et al.^69^

ATR-FTIR spectroscopy was used to investigate major shifts in cell physiology of *G. sulfurreducens*,^61,62^ and spectra for all assays suggested a pronounced change in the relative concentration of polysaccharides over time, indicated by changes in the bands at 1189–956 cm^–1^ (Figure 3). These changes were linked to glycogen synthesis, a polymer synthesized under limited growth conditions with excess carbon in the medium and serving as a dynamic energy-reserve for bacterial cells.^63,64^ An increase in the polysaccharide band for all assay conditions within the first 2 h indicated that cells responded to changes in the medium composition (cell transfer from the growth medium to the assay buffers) by net-synthesizing polysaccharides (Figure 3). After 2 h, a shift in metabolism was observed for all assays, and polysaccharides were net-consumed, suggesting that glycogen was utilized by the cells. The glycogen consumption increased among assays in the order standard assay < metabolite assay < nutrient assay. Glycogen utilization is known to provide cells short-term benefits under altering environmental conditions and facilitate improved nutrient uptake.^65^ Further, small increases in the relative content of nucleic acids, indicated by the bands at 1240–1220 cm^–1^, suggested that the cell number increased throughout the incubation time (Figure 3).^66^ Indeed, the measured cell density remained fairly constant during the first 6 h of incubation but increased from 6 to 24 h in the same order among treatments as observed for glycogen consumption and nucleic acid synthesis (Figure S2). The additional nutrients thus promoted an increased consumption of cellular polysaccharides (e.g., glycogen) at extended time intervals (≥6 h) in the nutrient assay compared to the metabolite and standard assay. This was accompanied by a doubling of the cell density in nutrient assays and a more modest increase in the metabolite assays during the 24 h.

Overall, the combined results of ATR-FTIR and cell density measurements showed that modest increases in nutrient availability stimulated cell metabolism and growth. As a consequence of higher metabolic activity, methylation in the nutrient assay was enhanced to similar levels as observed in the metabolite assay despite considerably lower concentrations of Hg(II) species with small thiols (as discussed above). Metabolic activity of microorganisms has been discussed as one of the major drivers for Hg(II) methylation in previous studies.^10,22,29,32,34,35^ Our study highlights that both enhanced Hg(II) availability and cell physiology increase Hg(II) methylation but that the responses are not necessarily additive since cell metabolism can largely impact the composition of ligands, like thiol compounds, binding Hg(II).

Our results also show that *G. sulfurreducens* cells can undergo substantial metabolic shifts and adaptation during the first 6 h when transferred from growth medium to washed cell assays (Figure 3). This time frame is typically used for Hg(II) methylation assays with bacteria cultures which means that methylation rates thus are commonly generated under conditions where cells can be expected to undergo metabolic shifts. The shift from net synthesis to net consumption of glycogen roughly coincided in time with the decrease in MeHg formation in our assays. We therefore carried out additional experiments with aged cells to specifically test if the cellular Hg(II) methylation capacity was maintained or not after this metabolic shift.

Cells were aged for 6 h (i.e., after the shift to net-consumption of glycogen and at the time point where MeHg formation normally plateaued, Figure 1, Figure 3) in Hg(II) free standard or metabolite assay buffer, and Hg(II) was added to a final concentration of 30 nM. We noted that the 6 h of cell aging did not lead to inhibition of MeHg formation. In the metabolite assay, the amount of MeHg formed 24 h after Hg(II) addition to nonaged and to 6 h aged cells was very similar (8.7 ± 0.9 nM as compared to 8.6 ± 0.8 nM MeHg, Figure S1b, Table S3, Table S7). In both cases, methylation progressed until the dissolved Hg(II) pool was depleted. In the standard assay, the amount of formed MeHg 24 h after Hg(II) addition was ∼60% higher in assays with 6 h aged, as compared to nonaged cells, 6.7 ± 0.4 nM and 4.0 ± 2.4 nM, respectively (Table S3, Table S7). In both cases, MeHg formation plateaued 6 h after the Hg(II) addition (corresponding to the 12 h time point for the aged cell assays in Figure S1a). Higher amounts of formed MeHg in the aged cell assays can be explained by differences in concentrations of small LMM-thiols (∼120 nM in 6 h aged assays compared to ∼30 nM in standard assays at 6.5 and 0.5 h respectively, Table S4, Table S8). Also, the very low concentrations of branched thiols in the 6 h aged cell assay buffers contributed to these differences (1–10 nM branched LMM-thiols, Table S8). Overall, these experiments showed that cells maintained their Hg(II) methylation capacity after 6 h of aging in the standard or metabolite assays. Assuming the same shift from net synthesis to net consumption of glycogen in aged cells (not measured), as in nonaged cells, our methylation results suggest that the metabolic shift in the time window from 2 to 6 h did not substantially impact Hg(II) methylation.

Since the metabolic capacity of *G. sulfurreducens* cells to methylate Hg(II) was maintained after 6 h, we further investigated if the addition of metabolite medium to the standard assay at 6 h (i.e., at the plateauing in MeHg formation) could restore methylation by causing a shift in speciation and/or partitioning of Hg(II). However, the addition of metabolite medium at 6 h did not cause an immediate increase in Hg(II) methylation (Figure S1c) despite a large increase in cysteine concentration (460 ± 200 nM). In these experiments, the speciation of dissolved Hg(II) was dominated by the Hg(Cys)_2_ complex both prior to, and after, the addition of metabolites. Thus, the added metabolite medium did not alter the distribution of Hg(II) between complexes with small and branched thiols in this case. Further, the enhanced cysteine concentrations following addition of the metabolite medium at 6 h did not alter the partitioning of Hg(II) between cellular and dissolved phase. This result was in contrast to the increased Hg(II) concentration in the dissolved phase when metabolites or cysteine alone was added to the buffer before the inoculation of cells (i.e., at the time point *t* = −1 h). Apparently, the addition of cysteine (∼450 nM) could not efficiently remobilize Hg(II) that had been sequestered by cells for 6 h. Previous studies with the ND132 bacterium have suggested that Hg(II) adsorbed/sequestered by cells can be remobilized and made available for methylation by the addition of thiols or dissolved organic matter resulting in increased MeHg formation.^20,23,25^ However, no remobilization of dissolved Hg(II) was observed in our experiments with *G. sulfurreducens* assays (Table S7). At extended times between 12 and 30 h, Hg(II) methylation was slightly increased in standard assays with metabolite medium added at 6 h compared to assays without metabolite addition, resulting in a cumulative MeHg concentration difference of ∼1.5 nM (Table S3, Table S7). It is possible that the added metabolites impacted the physiological state of cells by promoting metabolic activity and consequently increasing Hg(II) methylation.

Changes in the cell physiology are crucial in natural biofilms, since availability of nutrients and exposure to exogenous and endogenous metabolites vary depending on the spatial position within the biofilm and impact the growth of microorganism.^67,68^ However, our study showed that *G. sulfurreducens* maintained a similar capacity to methylate Hg(II) when shifting the physiological state from net-synthesis to net-consumption of glycogen.

### Environmental Implications

This study advances our understanding of how Hg(II) availability in terms of Hg(II) partitioning and speciation of dissolved Hg(II) and cell physiology govern microbial Hg(II) methylation. Our results show that cell physiology has multiple and complex effects on MeHg formation by controlling both the metabolic capacity of cells to methylate Hg(II) and the concentration of important ligands governing the speciation of Hg(II). Specifically, we demonstrate that bacterial metabolism of environmentally relevant concentrations of cysteine influences Hg(II) availability and methylation, in particular under conditions with added exogeneous cysteine. Enhanced Hg(II) methylation is observed under conditions where cysteine addition leads to an increased concentration of Hg(Cys)_2_ in solution. This can be achieved by increasing cysteine concentrations that shift the partitioning of Hg(II) from bacterial cell to the dissolved phase and/or change the speciation of dissolved Hg(II) from complexes with branched thiols to Hg(Cys)_2_ (these processes are indicated by 1 in the abstract figure). Added exogenous cysteine is, however, metabolized by *G. sulfurreducens* cells over time, and one important metabolite is penicillamine. This process leads to decreased Hg(II) availability and methylation due to a shift from Hg(II) complexes with small (cysteine and cysteamine) to branched (penicillamine) thiols (the shift over time from the left-hand to the right-hand condition in the abstract figure is indicated by 2). The effect of exogenous cysteine addition on Hg(II) methylation was therefore different among treatments (Figure 1) due to the cell physiology-dependent rate of metabolic conversion of cysteine to penicillamine (indicated by 3 in the abstract figure). In the environment, *G. sulfurreducens* is mainly present in biofilms, where cysteine and penicillamine are reported as two major LMM thiols.^27,28^ It is therefore plausible that the relative concentrations of these two compounds are a major control of Hg(II) availability and its methylation by *G. sulfurreducens* in environmental settings encountering low-sulfidic conditions. Our results highlight the need to carefully monitor the concentrations of biogenic produced and exogenous added LMM-thiol compounds, including penicillamine, in pure culture experiments, as well as in natural biofilms, when studying MeHg formation. Moreover, there is a need to identify metabolic features controlling the rate of microbial Hg(II) methylation. In agreement with previous studies, our results support that the Hg(II) methylation increases with nutrient availability and the overall metabolic activity of cells. However, our study also suggests that in environments with high exogeneous cysteine production, increased methylation capacity at high metabolic activity may be partly counteracted by a decline in cysteine and thereby Hg(II) availability. Our study further shows that *G. sulfurreducens* is capable of methylating Hg(II) at similar rates under metabolic phases of net-synthesis (energy-preservation) and net-consumption (glycogen utilization) of glycogen. Bacteria are expected to frequently undergo a dynamic physiological transition in nature (for example in biofilms) depending on nutrient availability. *G. sulfurreducens* cells are sensitive to nutrient fluctuation by adapting their metabolism and utilization of glycogen, but our results suggest that glycogen utilization *per se* will not lead to substantial variations in Hg(II) methylation potential.

## Supporting Information Available

Method text describing the Hg(II) methylation rate models; eight tables and five figures (PDF)
