# Supplementary material for: The Combined Effect of Hg(II) Speciation, Thiol Metabolism, and Cell Physiology on Methylmercury Formation by Geobacter sulfurreducens
Source: Environ Sci Technol. 2023 Apr 25;57(18):7185–95. doi: 10.1021/acs.est.3c00226 (PMC10173453; doi:10.1021/acs.est.3c00226)
Supplement: Supplementary file 1 — es3c00226_si_001.pdf [file es3c00226_si_001.pdf]

Supporting Information:

The combined effect of Hg(II) speciation, thiol metabolism and cell physiology on methylmercury formation by *Geobacter sulfurreducens*

*Mareike Gutensohn[1], Jeffra K. Schaefer[2], Elena Yunda[1], Ulf Skyllberg[3], Erik Björn[1]**

[1]Department of Chemistry, Umeå University, SE- 90187 Umeå, Sweden

[2]Department of Environmental Sciences, Rutgers University, 14 College Farm Road, New Brunswick, New Jersey 08901, United States

[3]Department of Forest Ecology and Management, Swedish University of Agricultural Sciences, SE-901 83 Umeå, Sweden

Number of pages: 17

Number of figures: 6

Number of tables: 8

Hg(II) species-specific, kinetic model for MeHg formation

We modeled the formation of MeHg by Hg(II) species-specific, first-order rate models. The models were based on the speciation of dissolved Hg(II) determined at the individual time points (0.5, 2, 6, and 24 h) of the assays. As described in the paper, speciation of dissolved Hg(II) was determined by thermodynamic modelling using the measured concentrations of dissolved Hg(II) and LMM-thiol compounds at each time point. We took advantage of this time-resolved Hg(II) speciation data by calculating the amount of MeHg formed for each time interval and subsequently calculating cumulative MeHg concentrations. The three dominant $Hg(LMM\text{-}RS)_2$ species $Hg(Cys)_2$, $Hg(CysN)_2$ and $Hg(PEN)_2$ were included in the model. The Hg(II) methylation rate constant, k_{meth}, for the $Hg(Cys)_2$ species was optimized by fitting calculated MeHg concentrations to measured concentrations. The k_{meth} values for the other two major Hg(II) complexes were calculated as dependent on k_{meth} values for $Hg(Cys)_2$: $k_{\text{meth}}(Hg(CysN)_2) = k_{\text{meth}}(Hg(Cys)_2)$ and $k_{\text{meth}}(Hg(PEN)_2) = 0.05 \times k_{\text{meth}}(Hg(Cys)_2)$. The relative differences of the sizes of the three rate constants are based on data from Schaefer et al. 2011 who (in *G. sulfurreducens* assays) determined k_{meth} values that were similar in size for $Hg(Cys)_2$ and $Hg(CysN)_2$ and approximately 20-fold lower for $Hg(PEN)_2$.[1] In our model, these rate constants are multiplied with the time-resolved measured cell density to account for differences in cell number changes, e.g. $k_{\text{meth}}(Hg(Cys)_2)$ (h^{-1}) = $k_{\text{meth}}(Hg(Cys)_2)$ (L $cell^{-1}$ h^{-1}) × cell density (cell L^{-1}). In each treatment, $k_{\text{meth}}(Hg(Cys)_2)$ was fitted independently by optimizing the residual sum of squares $(1 - \frac{\sum(y-\hat{y})^2}{\sum(y-\bar{y})^2})$ for the MeHg concentrations of the first three measurement time points.

$$[\mathrm{MeHg}] = \sum_{i=0}^{n} [\mathrm{Hg(Cys)_2}]_{\mathrm{initial}} \times (1 - \mathrm{e}^{-k_{\mathrm{meth}}(\mathrm{Hg(Cys)_2}) \times \Delta t})$$

$$+ \sum_{i=0}^{n} [\mathrm{Hg(CysN)_2}]_{\mathrm{initial}} \times (1 - \mathrm{e}^{-k_{\mathrm{meth}}(\mathrm{Hg(CysN)_2}) \times \Delta t})$$

$$+ \sum_{i=0}^{n} [\mathrm{Hg(PEN)_2}]_{\mathrm{initial}} \times (1 - \mathrm{e}^{-k_{\mathrm{meth}}(\mathrm{Hg(PEN)_2}) \times \Delta t})$$

The terms of the format $[\mathrm{Hg(LMM\text{-}RS)_2}]_{\mathrm{initial}}$ denote the concentration of the species at the start of each time-interval (*i*) and Δt is the duration of the interval in hours. For simplicity, it was assumed that the species distribution among the $\mathrm{Hg(LMM\text{-}RS)_2}$ complexes, and the cell density, changed linearly over time between two consecutive experimental data points. Due to the lack of measured values at t = 0 h the Hg(II) species concentrations were set equal at 0 and 0.5 h. In addition to changes in Hg(II) speciation and cell density, the modeled parameters also by necessity would reflect additional processes other than methylation that potentially affect the total concentration of dissolved Hg(II) in the assays (either by resupply or loss processes via e.g. changes in Hg(II) partitioning and reduction). For comparison, we therefore also applied a first-order rate model in which the measured total concentration of dissolved Hg(II), irrespective of its chemical speciation, $\mathrm{Hg(II)_{aq}}$, was considered as a substrate for bacterial methylation. For each assay condition, the methylation rate constant $k_{meth}(\mathrm{Hg(II)_{aq}})$ was optimized for the first three time intervals (*i*), similar to the species-specific model.

$$[\mathrm{MeHg}] = \sum_{i=0}^{n} [\mathrm{Hg(II)_{aq}}]_{\mathrm{initial}} \times (1 - \mathrm{e}^{-k_{\mathrm{meth}}(\mathrm{Hg(II)_{aq}}) \times \Delta t})$$

This model takes into account possible changes in cell density and concentration of total dissolved Hg(II) in the same way as the species-specific model, but it does not take into account changes in chemical speciation of dissolved Hg(II).

Table S1: Chemical composition of the different growth medium and assay buffer used.

Component	Unit	Standard growth medium	Iron depleted growth medium	Standard assay buffer	Metabolite assay buffer*	Nutrient assay buffer
**MOPS**	mM	10	10	10	10	10
**NH_4Cl**	mM	5.6	5.6	0.1	0.1	0.7
**KCl**	mM	1.3	1.3	1.3	1.3	1.3
**$CaCl_2$**	µM	8.0	8.0	-	0.8	0.8
**NaCl**	mM	0.17	0.17	0.17	0.17	0.17
**$MgSO_4$**	mM	0.12	0.12	0.15	0.15	0.15
**NaH_2PO_4**	mM	0.05	0.05	5.00	4.51 - 5.00	4.51
**Acetate**	mM	10	10	1.0	1.0 - 1.9	1.9
**Fumarate**	mM	40	40	1.0	1.0 - 3.9	3.9
**Resazurin**	µM	4.0	4.0	4.0	4.0	4.0
**NTA**	µM	78.6	-	-	0 -10	7.86
**EDTA**	µM	-	100	-	0 -10	-
**$CoCl_2$**	µM	4.24	4.24	-	0 - 0.42	0.42
**$CuSO_4$**	µM	0.04	-	-	-	0.00
**$MnCl_2$**	µM	29.8	29.8	-	0 - 2.98	2.98
**$AlK(SO4)_2$**	µM	0.22	0.22	-	0 - 0.02	0.02
**H_3BO_3**	µM	1.60	1.60	-	0 - 0.16	0.16
**Na_2MoO_4**	µM	0.42	0.42	-	0 - 0.04	0.04
**$NiCl_2$**	µM	0.44	0.44	-	0 - 0.04	0.04
**$ZnSO_4$**	µM	3.48	3.48	-	0 -0.35	0.35
**$FeSO_4$**	µM	3.60	1.50	-	0 - 0.15	0.36
**Na_2SeO_3**	µM	0.6	0.6	-	0 - 0.06	0.06

*the concentration ranges given for the metabolite assay buffer reflects that this assay contained 10% Iron depleted growth medium which was isolated (bacteria cells were removed) after growing cells to the late exponential growth phase (~ 40h). The exact consumption of growth medium components during cell growth is not known and the given concentration ranges corresponds to 100% to 0% consumption.

Table S2: Species-Specific Methylation Rate Constants (k_{meth}, × 10^{-12} L $cell^{-1}$ h^{-1}) for $Hg(Cys)_2$

Cysteine	Incubation time	Standard assay	Metabolite assay	Nutrient assay	Reference
(nM)	(h)	k_{meth} (× 10^{-12} L $cell^{-1}$ h^{-1})			
0	6	0.70	0.58	3.3	This study
100		0.51	0.88	-	
600		1.1	1.6	0.85	
0	2	0.1 ± 0.4			Schaefer et al. (2011) [1]
10000		1.6 ± 0.5			
0	1	0.21 ± 0.7			Schaefer et al. (2014) [2]
0-100	6	0.34 ± 0.15			Adedrian et al. (2019) [3]

Table S3: Concentrations (nM) of different Hg fraction in standard, metabolite and nutrient assays with exogenous cysteine of 0, 100 and 600 nM after 0.5, 2, 6 and 24 h incubation time. Total Hg, total Hg(diss) and total MeHg concentrations are based on measurements. Calculation of the different Hg fraction: Total cellular Hg = Total Hg – Total dissolved Hg; Total Hg losses = added Hg – Total Hg; dissolved MeHg = partitioning factor × total MeHg; cellular MeHg = Total MeHg – dissolved MeHg; Hg(II) = Total Hg – Total MeHg; dissolved Hg(II) = Total dissolved Hg – dissolved MeHg; cellular Hg(II) = Total cellular Hg – cellular MeHg. (n=3; ± standard deviation)

	**Time** (h)	**Total Hg** (nM)	**Total Hg(diss)** (nM)	**Total Hg (cell-associated)** (nM)	**Total Hg losses** (nM)	**Total MeHg** (nM)	**Fraction dissolved MeHg***	**MeHg (diss)** (nM)	**MeHg (cell-associated)** (nM)	**Hg(II)** (nM)	**Hg(II) (diss)** (nM)	**Hg(II) (cell-associated)** (nM)
**Standard assay**												
0 nM cysteine	0.5	19 ± 2.3	9.2 ± 0.6	10 ± 2.8	11 ± 2.3	0.8 ± 0.3	0.17	0.1 ± 0.1	0.6 ± 0.3	19 ± 2.4	9.0 ± 0.6	9.6 ± 3.1
	2	18 ± 2.3	9.5 ± 0.6	8.4 ± 3.0	12 ± 2.3	2.0 ± 0.9	0.24	0.5 ± 0.2	1.5 ± 0.7	16 ± 2.0	9.1 ± 0.9	6.9 ± 3.6
	6	16 ± 1.9	9.1 ± 0.6	7.0 ± 2.5	14 ± 1.9	3.5 ± 2.2	0.20	0.7 ± 0.4	2.8 ± 1.8	13 ± 1.4	8.4 ± 1.0	4.1 ± 4.2
	24	15 ± 1.6	8.9 ± 1.0	6.3 ± 2.6	15 ± 1.6	4.0 ± 2.4	0.75	3.0 ± 1.8	1.0 ± 0.6	11 ± 1.9	5.9 ± 2.8	5.3 ± 3.2
100 nM cysteine	0.5	23 ± 2.3	14 ± 0.8	8.7 ± 3.1	6.9 ± 2.3	1.2 ± 0.6	0.24	0.3 ± 0.1	0.9 ± 0.4	22 ± 1.8	14 ± 0.9	7.9 ± 3.6
	2	22 ± 2.0	15 ± 1.7	6.7 ± 3.7	8.5 ± 2.0	2.6 ± 0.8	0.28	0.7 ± 0.2	1.9 ± 0.6	19 ± 1.2	14 ± 1.9	4.9 ± 4.3
	6	20 ± 2.4	14 ± 2.1	6.2 ± 4.5	10 ± 2.4	3.9 ± 0.7	0.22	0.8 ± 0.1	3.0 ± 0.5	16 ± 2.1	13 ± 2.2	3.2 ± 5.1
	24	18 ± 4.0	12 ± 3.0	5.5 ± 7.0	12 ± 4.0	4.1 ± 0.7	0.75	3.1 ± 0.5	1.0 ± 0.2	14 ± 3.5	9.2 ± 3.5	4.4 ± 7.2
600 nM cysteine	0.5	24 ± 0.0	11 ± 1.4	12 ± 1.5	6.5 ± 0.0	1.3 ± 0.4	0.74	1.0 ± 0.3	0.3 ± 0.1	22 ± 0.4	10 ± 1.7	12 ± 1.6
	2	22 ± 0.7	14 ± 0.2	7.4 ± 0.9	8.3 ± 0.7	3.2 ± 0.8	0.67	2.1 ± 0.5	1.1 ± 0.3	19 ± 0.1	12 ± 0.7	6.3 ± 1.2
	6	22 ± 1.5	13 ± 0.6	7.1 ± 2.2	9.4 ± 1.5	3.8 ± 0.4	0.47	1.8 ± 0.2	2.0 ± 0.2	17 ± 1.1	12 ± 0.8	5.2 ± 2.4
	24	18 ± 2.9	12 ± 1.4	6.0 ± 4.3	12 ± 2.9	5.0 ± 0.6	0.75	3.7 ± 0.5	1.2 ± 0.2	13 ± 3.5	8.1 ± 1.8	4.8 ± 4.4
**Metabolite assay**												
0 nM cysteine	0.5	25 ± 2.8	19 ± 2.7	6.6 ± 5.5	4.6 ± 2.8	1.0 ± 0.2	0.24	0.2 ± 0.0	0.7 ± 0.1	25 ± 2.8	19 ± 2.7	5.8 ± 5.7
	2	24 ± 2.3	16 ± 2.1	7.5 ± 4.5	6.1 ± 2.3	3.1 ± 0.1	0.24	0.8 ± 0.0	2.4 ± 0.1	21 ± 2.4	16 ± 2.1	5.1 ± 4.5
	6	22 ± 2.8	15 ± 2.0	7.0 ± 4.8	8.4 ± 2.8	5.9 ± 0.4	0.24	1.4 ± 0.1	4.5 ± 0.3	16 ± 2.5	13 ± 2.1	2.5 ± 5.1
	24	21 ± 5.4	13 ± 3.2	8.4 ± 8.6	8.8 ± 5.4	8.6 ± 0.8	0.75	6.4 ± 0.6	2.1 ± 0.2	12 ± 4.3	6.4 ± 3.8	6.3 ± 8.8
100 nM cysteine	0.5	24 ± 1.4	17 ± 1.9	6.9 ± 3.3	5.7 ± 1.4	1.0 ± 0.1	0.83	0.8 ± 0.1	0.2 ± 0.0	23 ± 1.4	17 ± 2.0	6.8 ± 3.3
	2	23 ± 2.2	14 ± 2.0	9.1 ± 4.2	7.0 ± 2.2	3.5 ± 0.9	0.78	2.7 ± 0.7	0.8 ± 0.2	20 ± 1.6	11 ± 2.7	8.4 ± 4.4
	6	20 ± 3.0	12 ± 1.9	8.2 ± 4.9	9.7 ± 3.0	7.4 ± 3.5	0.34	2.6 ± 1.2	4.9 ± 2.3	13 ± 2.3	9.5 ± 3.1	3.4 ± 7.2
	24	19 ± 3.8	8.5 ± 2.5	10 ± 6.3	11 ± 3.8	10 ± 4.1	0.75	7.7 ± 3.1	2.6 ± 1.0	8.4 ± 0.9	0.9 ± 5.6	7.5 ± 7.3
600 nM cysteine	0.5	26 ± 4.2	16 ± 1.1	10 ± 5.3	3.8 ± 4.2	1.3 ± 0.4	0.90	1.2 ± 0.4	0.1 ± 0.0	25 ± 3.8	15 ± 1.5	10.3 ± 5.4
	2	24 ± 4.3	12 ± 2.0	13 ± 6.3	6.1 ± 4.3	3.6 ± 1.1	0.90	3.3 ± 1.0	0.4 ± 0.1	20 ± 3.2	8.2 ± 3.0	12.1 ± 6.4
	6	22 ± 4.7	9.7 ± 2.6	13 ± 7.2	7.8 ± 4.7	9.5 ± 5.3	0.90	8.6 ± 4.8	0.9 ± 0.5	13 ± 2.4	1.1 ± 7.3	11.6 ± 7.8
	24	20 ± 4.3	8.7 ± 1.4	11 ± 5.7	9.9 ± 4.3	12 ± 6.8	0.75	8.6 ± 5.1	2.9 ± 1.7	8.6 ± 4.1	0.1 ± 6.5	8.5 ± 7.4
**Nutrient assay**												
0 nM cysteine	0.5	23 ± 1.2	10 ± 1.4	13 ± 2.6	6.7 ± 1.2	1.1 ± 0.2	0.17	0.2 ± 0.0	0.9 ± 0.2	22 ± 1.0	9.8 ± 1.4	12 ± 2.8
	2	22 ± 1.1	9.7 ± 1.3	13 ± 2.4	7.7 ± 1.1	2.7 ± 0.3	0.22	0.6 ± 0.1	2.1 ± 0.2	20 ± 1.0	9.1 ± 1.3	10 ± 2.6
	6	21 ± 1.4	8.7 ± 0.6	11 ± 2.1	9.5 ± 1.4	6.3 ± 0.9	0.17	1.1 ± 0.2	5.2 ± 0.7	14 ± 0.7	7.7 ± 0.8	6.5 ± 2.8
	24	18 ± 0.8	8.0 ± 1.0	10 ± 1.7	12 ± 0.8	7.8 ± 1.2	0.75	5.9 ± 0.9	2.0 ± 0.3	11 ± 0.5	2.1 ± 1.9	8.5 ± 2.0
100 nM cysteine	0.5	24 ± 4.3	13 ± 1.7	11 ± 6.0	5.7 ± 4.3	1.5 ± 0.1	0.17	0.3 ± 0.0	1.2 ± 0.1	23 ± 4.4	13 ± 1.7	10 ± 6.1
	2	24 ± 3.9	15 ± 2.4	9.1 ± 6.3	5.8 ± 3.9	2.8 ± 0.2	0.24	0.7 ± 0.1	2.2 ± 0.2	21 ± 4.0	14 ± 2.4	7.0 ± 6.5
	6	23 ± 3.4	15 ± 2.7	8.2 ± 6.2	6.9 ± 3.4	3.5 ± 0.7	0.19	0.7 ± 0.1	2.8 ± 0.6	30 ± 3.5	14 ± 2.9	5.4 ± 6.8
	24	22 ± 3.8	14 ± 3.1	8.0 ± 6.8	8.0 ± 3.8	4.0 ± 0.3	0.75	3.0 ± 0.2	1.0 ± 0.1	18 ± 3.8	11 ± 3.3	7.0 ± 6.9
600 nM cysteine	0.5	25 ± 1.5	12 ± 2.1	12 ± 3.5	5.4 ± 1.5	1.6 ± 0.5	0.45	0.7 ± 0.2	0.9 ± 0.3	23 ± 1.7	12 ± 2.3	12 ± 3.8
	2	23 ± 1.2	14 ± 2.4	8.9 ± 3.6	7.2 ± 1.2	3.3 ± 1.1	0.19	0.6 ± 0.2	2.7 ± 0.9	19 ± 1.9	13 ± 2.6	6.2 ± 4.5
	6	21 ± 1.1	13 ± 1.7	7.5 ± 2.8	9.3 ± 1.1	3.9 ± 1.3	0.22	0.8 ± 0.3	3.0 ± 1.0	17 ± 0.8	12 ± 1.9	4.5 ± 3.8
	24	18 ± 1.1	11 ± 0.9	6.6 ± 2.0	12 ± 1.1	5.0 ± 1.7	0.75	3.7 ± 1.3	1.2 ± 0.4	13 ± 0.7	7.6 ± 2.1	5.4 ± 2.4

* The fraction of dissolved MeHg is based on the assumption of a linear relationship between the dissolved concentration of LMM-RSH and fraction of MeHg in the dissolved phase based on data in Figure 2a by Lin et al. 2015.[4] Calculation of the fraction of dissolved MeHg for LMM-RSH concentration between 0 and 1000 nM: $f(MeHg(dissolved)) = 7.7\times10^{-4} \times \sum[LMM\text{-}RSH] + 0.13$, and for LMM-RSH concentration and between 1000 and 50000 nM: $f(MeHg(dissolved)) = 2.04\times10^{-6} \times \sum[LMM\text{-}RSH] + 0.9$, where ∑[LMM-RSH] is the measured sum concentration of dissolved LMM-RSH compounds.

Table S4: LMM-thiol concentrations (nM) in standard, metabolite and nutrient assays with exogenous cysteine of 0, 100 and 600 nM after 0.5, 2, 6 and 24 h incubation time (n=3; ± standard deviation).[a] The LMM-thiol concentration data was reproduced from Gutensohn et al..[5]

	Time	Cys	CysN	HCys	Glyc	MAC	Σ small LMM-RSH	PEN	NacCys	NacPEN	Σ branched LMM-RSH	Σ LMM-RSH
	(h)	(nM)	(nM)	(nM)	(nM)	(nM)	(nM)	(nM)	(nM)	(nM)	(nM)	(nM)
**Standard assay**												
0 nM cysteine	0.5	3.3 ± 5.7	25 ± 15	5.3 ± 9.2	-	-	34 ± 22	5.6 ± 6.0	4.0 ± 6.9	2.7 ± 4.7	12 ± 17	46
	2	3.4 ± 5.9	130 ± 180	5.2 ± 9.0	-	-	130 ± 180	5.4 ± 5.3	4.8 ± 8.3	2.7 ± 4.7	13 ± 18	150
	6	3.2 ± 5.6	64 ± 61	5.2 ± 9.0	-	-	73 ± 57	4.0 ± 5.7	10 ± 18	2.8 ± 4.8	17 ± 28	90
	24	6.8 ± 7.9	49 ± 54	6.9 ± 8.2	0.03 ± 0.1	2.3 ± 4.0	65 ± 62	19 ± 18	15 ± 20	2.8 ± 4.9	37 ± 29	100
100 nM cysteine	0.5	77 ± 120	64 ± 77	-	-	-	140 ± 74	8.2 ± 8.3	-	-	8.2 ± 8.3	150
	2	37 ± 25	136 ± 210	10 ± 8.8	-	-	180 ± 240	11 ± 10	-	-	11 ± 10	190
	6	7.7 ± 7.3	43 ± 52	5.2 ± 9.1	-	-	55 ± 46	57 ± 88	-	-	57 ± 88	110
	24	9.1 ± 8.7	31 ± 40	1.6 ± 2.8	0.03 ± 0.1	2.2 ± 3.7	44 ± 50	61 ± 43	3.5 ± 6.0	-	65 ± 45	110
600 nM cysteine	0.5	670 ± 830	24 ± 17	5.3 ± 9.1	-	-	700 ± 840	43 ± 56	45 ± 66	2.7 ± 4.7	90 ± 120	790
	2	210 ± 300	130 ± 190	150 ± 240	-	-	490 ± 720	190 ± 300	15 ± 14	2.7 ± 4.7	210 ± 310	690
	6	12 ± 11	35 ± 33	-	-	-	47 ± 42	390 ± 600	7.6 ± 8.0	2.7 ± 4.6	400 ± 600	440
	24	14 ± 12	6.8 ± 12	1.6 ± 0.0	0.03 ± 0.0	2.1 ± 0.0	24 ± 21	150 ± 35	11 ± 14	2.8 ± 4.8	160 ± 150	190
**Metabolite assay**												
0 nM cysteine	0.5	91 ± 50	23 ± 40	11 ± 19	0.4 ± 0.6	-	130 ± 77	16 ± 4.9	-	-	16 ± 4.9	140
	2	79 ± 99	20 ± 18	11 ± 18	16 ± 28	-	130 ± 160	17 ± 12	-	-	17 ± 12	140
	6	59 ± 100	25 ± 11	10 ± 18	4.5 ± 7.8	-	98 ± 140	48 ± 47	-	-	48 ± 47	150
	24	16 ± 28	16 ± 7.5	11 ± 19	-	-	44 ± 53	340 ± 370	-	-	340 ± 370	380
100 nM cysteine	0.5	750 ± 810	66 ± 42	-	-	-	890 ± 780	8.8 ± 7.6	6.1 ± 10	2.9 ± 5.1	19 ± 20	910
	2	800 ± 970	21 ± 23	-	-	-	820 ± 960	9.1 ± 7.9	6.6 ± 11	2.8 ± 4.9	18 ± 22	840
	6	130 ± 220	39 ± 68	-	-	-	170 ± 190	95 ± 110	6.1 ± 10	2.7 ± 4.7	100 ± 100	280
	24	87 ± 83	32 ± 56	-	-	-	220 ± 310	670 ± 640	20.2 ± 18	2.8 ± 4.8	310 ± 54	530
600 nM cysteine	0.5	1100 ± 580	14 ± 24	20 ± 17	10 ± 12	-	1200 ± 620	31 ± 29	-	-	31 ± 29	1200
	2	1500 ± 1000	130 ± 140	21 ± 18	-	-	1700 ± 890	37 ± 32	2.0 ± 3.4	-	39 ± 29	1700
	6	3900 ± 310	35 ± 28	21 ± 18	3.7 ± 6.4	-	450 ± 360	690 ± 230	2.7 ± 3.2	-	690 ± 230	1100
	24	110 ± 53	8.0 ± 14	21 ± 18	-	-	217 ± 160	950 ± 1100	2.6 ± 4.4	-	380 ± 290	590
**Nutrient assay**												
0 nM cysteine	0.5	11 ± 18	0.2 ± 0.4	10 ± 18	5.7 ± 10	-	27 ± 46	24 ± 22	4.1 ± 7.1	2.7 ± 4.6	30 ± 20	57
	2	9.7 ± 16	60 ± 100	9.9 ± 17	1.3 ± 2.3	-	81 ± 91	27 ± 32	4.1 ± 7.1	2.7 ± 4.6	34 ± 28	120
	6	8.8 ± 15	5.5 ± 9.4	9.9 ± 17	3.3 ± 5.7	-	28 ± 48	23 ± 22	5.2 ± 9.0	2.7 ± 4.6	31 ± 20	58
	24	63 ± 110	6.9 ± 7.7	9.9 ± 17	1.2 ± 2.1	-	81 ± 130	270 ± 400	12 ± 17	2.7 ± 4.7	290 ± 400	370
100 nM cysteine	0.5	7.5 ± 6.5	25 ± 22	5.2 ± 9.1	-	-	38 ± 34	7.1 ± 3.9	4.3 ± 7.4	2.7 ± 4.6	14 ± 16	52
	2	8.3 ± 7.2	110 ± 110	10 ± 9.0	-	-	130 ± 92	9.3 ± 1.6	4.4 ± 7.6	2.7 ± 4.6	16 ± 14	140
	6	7.9 ± 6.8	49 ± 28	5.2 ± 9.1	0.2 ± 0.3	-	62 ± 21	8.4 ± 5.3	4.6 ± 8.0	2.7 ± 4.6	16 ± 15	78
	24	8.4 ± 7.3	19 ± 19	11 ± 9.1	-	-	38 ± 34	34 ± 18	9.4 ± 16	2.7 ± 4.7	46 ± 39	84
600 nM cysteine	0.5	350 ± 480	40 ± 37	5.2 ± 9.0	-	-	400 ± 440	8.6 ± 3.0	4.6 ± 7.9	2.7 ± 4.6	16 ± 15	420
	2	11.6 ± 16	38 ± 33	-	-	-	50 ± 45	24 ± 17	4.4 ± 7.5	2.7 ± 4.6	31 ± 14	81
	6	9.0 ± 16	18 ± 25	5.3 ± 9.2	0.9 ± 1.6	-	34 ± 51	72 ± 73	4.9 ± 8.4	2.7 ± 4.6	79 ± 67	110
	24	5.0 ± 8.7	17 ± 15	-	-	-	22 ± 20	110 ± 73	11 ± 19	2.7 ± 4.7	120 ± 78	140

[a] Cells marked with "-" when values are under detection limit.

Table S5: Concentrations (nM) of the different Hg(II) species in the extracellular buffer of *G.sulfurreducens* in standard, metabolite and nutrient assays with exogenous cysteine of 0, 100 and 600 nM after 0.5, 2, 6 and 24 h incubation time. Thermodynamic model was performed by using the software WinSGW. [6, b]

	Time	$Hg(Cys)_2$	$Hg(CysN)_2$	$Hg(HCys)_2$	$Hg(Glyc)_2$	$Hg(MAC)_2$	Σ small $Hg(LMM\text{-}RS)_2$	$Hg(PEN)_2$	$Hg(NacCys)_2$	$Hg(NacPEN)_2$	Σ branched $Hg(LMM\text{-}RS)_2$	additional Hg(II) species
	(h)	(nM)	(nM)	(nM)	(nM)	(nM)	(nM)	(nM)	(nM)	(nM)	(nM)	(nM)
**Standard assay**												
0 nM cysteine	0.5	0.44	5.30	0.44	-	-	6.18	1.01	0.93	0.68	2.62	0.20
	2	0.03	8.77	0.02	-	-	8.81	0.07	0.13	0.08	0.28	0.01
	6	0.07	6.66	0.06	-	-	6.80	0.10	1.26	0.22	1.58	0.03
	24	-	0.04	-	-	-	0.04	5.82	0.03	-	5.85	0.01
100 nM cysteine	0.5	10.3	3.39	-	-	-	13.7	0.23	-	-	0.23	0.05
	2	2.63	10.9	0.08	-	-	13.6	0.33	-	-	0.33	0.03
	6	0.31	2.69	0.05	-	-	3.06	9.80	-	-	9.80	0.14
	24	0.26	0.95	-	-	0.04	1.25	7.76	0.10	-	7.86	0.10
600 nM cysteine	0.5	9.82	0.00	-	0.01	-	9.83	0.05	0.12	-	0.17	-
	2	5.43	0.72	0.80	-	-	6.95	4.95	0.08	-	5.03	0.02
	6	0.01	0.03	-	-	-	0.04	11.9	0.01	-	11.9	0.02
	24	0.07	0.01	0.00	-	-	0.08	7.84	0.13	0.01	7.99	0.03
**Metabolite assay**												
0 nM cysteine	0.5	16.9	0.75	0.16	-	-	17.8	1.07	-	-	1.07	0.09
	2	13.9	0.61	0.16	-	-	14.7	1.21	-	-	1.21	0.09
	6	10.9	1.28	0.23	-	-	12.5	0.46	-	-	0.46	0.09
	24	0.01	-	-	-	-	0.02	6.37	-	-	6.37	0.01
100 nM cysteine	0.5	17.0	0.04	-	-	-	17.0	-	-	-	0.01	-
	2	11.0	-	-	-	-	11.0	-	-	-	-	-
	6	5.88	0.19	-	-	-	6.06	3.37	0.04	-	3.41	0.03
	24	0.18	0.01	-	-	-	0.19	0.69	-	-	0.70	-
600 nM cysteine	0.5	15.0	-	-	-	-	15.0	0.01	-	-	0.01	-
	2	8.17	0.02	-	-	-	8.19	0.01	-	-	0.01	-
	6	0.25	-	-	-	-	0.25	0.85	-	-	0.85	-
	24	0.02	-	-	-	-	0.02	0.09	-	-	0.09	-
**Nutrient assay**												
0 nM cysteine	0.5	1.56	0.00	0.64	0.96	-	3.16	5.31	0.62	0.45	6.38	0.26
	2	0.55	4.41	0.19	0.03	-	5.18	3.37	0.25	0.19	3.82	0.10
	6	0.99	0.18	0.50	0.34	-	2.01	4.35	0.76	0.39	5.49	0.20
	24	0.10	-	-	-	-	0.10	1.99	0.01	0.00	2.00	-
100 nM cysteine	0.5	2.71	2.77	1.38	-	-	6.86	2.29	1.69	1.07	5.05	1.09
	2	0.34	12.6	0.17	-	-	13.1	0.43	0.24	0.16	0.83	0.04
	6	1.12	9.91	0.24	-	-	11.3	1.24	0.83	0.50	2.57	0.17
	24	0.88	0.00	0.54	-	-	1.42	7.11	1.84	0.39	9.34	0.24
600 nM cysteine	0.5	10.9	0.05	-	-	-	11.0	0.01	0.01	0.00	0.02	0.01
	2	1.59	5.37	-	-	-	6.96	4.83	0.59	0.40	5.82	0.22
	6	0.28	0.30	0.03	0.01	-	0.62	11.0	0.20	0.12	11.3	0.12
	24	0.02	0.07	-	-	-	0.08	7.24	0.21	0.03	7.47	0.04

[b] Cells marked with "-" when values are < 0.01 nM

Table S6: Overview of the species, reactions, stability constants (logB) and reference of logB for the speciation model of Hg(II)(dissolved) in the extracellular assay buffer. The model matrix present the "species" as the reaction products of the "components". For instance, the chemical reaction: H2O = OH- + H+, log *K* = -13.7 is insert into the matrix as the species "OH-" with H2O - H+ = OH-. Cysteine (Cys), cysteamine (CysT), mercaptoacetic acid (MAC), monothioglycerol (Glyc), Homocysteine (HCys), Penicillamine (PEN), N-Acetyl-cysteine (NacCys), N-Acetyl-penicillamine (NacPEN).

Reference	Species	log *K*	H_2O	H+	Hg^{2+}	Cl^-	H_2S	Cys^-	MAC^-	PEN^-	Gly^-	$HCys^-$	$NacCys^-$	$CysN^-$	$NacPEN^-$	$R\text{-}COO^-$
7	OH-	-13.7	1	-1	0	0	0	0	0	0	0	0	0	0	0	0
8	HS-	-7	0	-1	0	0	1	0	0	0	0	0	0	0	0	0
9	Cys-H	8.64	0	1	0	0	0	1	0	0	0	0	0	0	0	0
9	MAC-H	10.16	0	1	0	0	0	0	1	0	0	0	0	0	0	0
9	PEN-H	8.3	0	1	0	0	0	0	0	1	0	0	0	0	0	0
9	Glyc-H	9.42	0	1	0	0	0	0	0	0	1	0	0	0	0	0
9	HCys-H	9.87	0	1	0	0	0	0	0	0	0	1	0	0	0	0
9	NACCys-H	9.79	0	1	0	0	0	0	0	0	0	0	1	0	0	0
9	CysT-H	10.3	0	1	0	0	0	0	0	0	0	0	0	1	0	0
9	NacPEN-H	9.6	0	1	0	0	0	0	0	0	0	0	0	0	1	0
10	RCOO-H	3.7	0	1	0	0	0	0	0	0	0	0	0	0	0	1
7	$HgOH^+$	-3.4	1	-1	1	0	0	0	0	0	0	0	0	0	0	0
7	$Hg(OH)_2$	-6.2	2	-2	1	0	0	0	0	0	0	0	0	0	0	0
7	$HgCl^+$	7.1	0	0	1	1	0	0	0	0	0	0	0	0	0	0
7	$HgCl_2$	13.8	0	0	1	2	0	0	0	0	0	0	0	0	0	0
7	$HgCl_3^-$	14.7	0	0	1	3	0	0	0	0	0	0	0	0	0	0
7	$HgCl_4^{2-}$	15.4	0	0	1	4	0	0	0	0	0	0	0	0	0	0
7	HgOHCl	4.3	1	-1	1	1	0	0	0	0	0	0	0	0	0	0
11	$HgSH^+$	13	0	-1	1	0	1	0	0	0	0	0	0	0	0	0
11	HgS_2H^-	18.2	0	-3	1	0	2	0	0	0	0	0	0	0	0	0
11	HgS_2^{2-}	8.9	0	-4	1	0	2	0	0	0	0	0	0	0	0	0
12	$Hg(SH)_2^0$	24.6	0	-2	1	0	2	0	0	0	0	0	0	0	0	0
11	HgOHSH	9.4	1	-2	1	0	1	0	0	0	0	0	0	0	0	0
11	HgClSH	18.9	0	-1	1	1	1	0	0	0	0	0	0	0	0	0
9	$Hg(Cys)_2$	37.5	0	0	1	0	0	2	0	0	0	0	0	0	0	0
9	$Hg(MAC)_2$	40.9	0	0	1	0	0	0	2	0	0	0	0	0	0	0
9	$Hg(PEN)_2$	36.9	0	0	1	0	0	0	0	2	0	0	0	0	0	0
9	$Hg(Glyc)_2$	39.4	0	0	1	0	0	0	0	0	2	0	0	0	0	0
9	$Hg(HCys)_2$	39.4	0	0	1	0	0	0	0	0	0	2	0	0	0	0
9	$Hg(NacCys)_2$	40.2	0	0	1	0	0	0	0	0	0	0	2	0	0	0
9	$Hg(CysN)_2$	40.3	0	0	1	0	0	0	0	0	0	0	0	2	0	0
9	$Hg(NacPEN)_2$	40.1	0	0	1	0	0	0	0	0	0	0	0	0	2	0
9, 13, 14	$HgCys^+$	25	0	0	1	0	0	1	0	0	0	0	0	0	0	0
9, 13, 14	$HgMAC^+$	26.5	0	0	1	0	0	0	1	0	0	0	0	0	0	0
9, 13, 14	$HgPEN^+$	25.7	0	0	1	0	0	0	0	1	0	0	0	0	0	0
9, 13, 14	$HgGlyc^+$	26	0	0	1	0	0	0	0	0	1	0	0	0	0	0
9, 13, 14	$HgHCys^+$	25.5	0	0	1	0	0	0	0	0	0	1	0	0	0	0
9, 13, 14	$HgNacCys^+$	26	0	0	1	0	0	0	0	0	0	0	1	0	0	0
9, 13, 14	$HgCysN^+$	25.7	0	0	1	0	0	0	0	0	0	0	0	1	0	0
9, 13, 14	$HgNacPEN^+$	26	0	0	1	0	0	0	0	0	0	0	0	0	1	0
9, 15	$Hg(Cys)_3$	40.45	0	0	1	0	0	3	0	0	0	0	0	0	0	0
9, 15	$Hg(Cys)_4$	42.6	0	0	1	0	0	4	0	0	0	0	0	0	0	0
11	HgOH(Cys)	18.5	1	-1	1	0	0	1	0	0	0	0	0	0	0	0
11	HgOH(MAC)	18.5	1	-1	1	0	0	0	1	0	0	0	0	0	0	0
11	HgOH(PEN)	18.5	1	-1	1	0	0	0	0	1	0	0	0	0	0	0
11	HgOH(Glyc)	18.5	1	-1	1	0	0	0	0	0	1	0	0	0	0	0
11	HgOH(Hcys)	18.5	1	-1	1	0	0	0	0	0	0	1	0	0	0	0
11	HgOH(NacCys)	18.5	1	-1	1	0	0	0	0	0	0	0	1	0	0	0
11	HgOH(CysN)	18.5	1	-1	1	0	0	0	0	0	0	0	0	1	0	0
11	HgOH(NacPEN)	18.5	1	-1	1	0	0	0	0	0	0	0	0	0	1	0
11	HgCl(Cys)	28.5	0	0	1	1	0	1	0	0	0	0	0	0	0	0
11	HgCl(MAC)	28.5	0	0	1	1	0	0	1	0	0	0	0	0	0	0
11	HgCl(PEN)	28.5	0	0	1	1	0	0	0	1	0	0	0	0	0	0
11	HgCl(Glyc)	28.5	0	0	1	1	0	0	0	0	1	0	0	0	0	0
11	HgCl(Hcys)	28.5	0	0	1	1	0	0	0	0	0	1	0	0	0	0
11	HgCl(NacCys)	28.5	0	0	1	1	0	0	0	0	0	0	1	0	0	0
11	HgCl(CysN)	28.5	0	0	1	1	0	0	0	0	0	0	0	1	0	0
11	HgCl(NacPEN)	28.5	0	0	1	1	0	0	0	0	0	0	0	0	1	0
16	$HgS_{(s)}$	29.8	0	-2	1	0	1	0	0	0	0	0	0	0	0	0

Table S7: Concentrations (nM) of different Hg fraction in three additional assay systems: (a) aged cells in standard assay, (b) aged cells in metabolite assay and (c) metabolite addition after 6 h in standard assay at different time points (h). Total Hg, total Hg(diss) and total MeHg concentrations are based on measurements. Calculation of the different Hg fraction: Total cellular Hg = Total Hg – Total dissolved Hg; Total Hg losses = added Hg – Total Hg; dissolved MeHg = partitioning factor × Total MeHg; cellular MeHg = Total MeHg – dissolved MeHg; Hg(II) = Total Hg – Total MeHg; dissolved Hg(II) = Total dissolved Hg – dissolved MeHg; cellular Hg(II) = Total cellular Hg – cellular MeHg. (n=3; ± standard deviation)

	**Time**	**Total Hg**	**Total Hg(diss)**	**Total Hg(cell-associated)**	**Total Hg losses**	**Total MeHg**	**MeHg(diss)**	**MeHg(cell-associated)**	**Hg(II)**	**Hg(II)(diss)**	**Hg(II) (cell-associated)**
	(h)	(nM)	(nM)	(nM)	(nM)	(nM)	(nM)	(nM)	(nM)	(nM)	(nM)
**(a) Aged cells in**	6.5	30 ± 1.9	21 ± 2.2	9.5 ± 4.1	0.9 ± 1.9	1.6 ± 0.1	0.4 ± 0.0	1.2 ± 0.1	29 ± 2.0	20 ± 2.1	8.3 ± 4.1
**standard assay,**	8	26 ± 0.2	18 ± 0.9	8.2 ± 1.1	3.7 ± 0.2	4.0 ± 0.3	1.1 ± 0.1	3.0 ± 0.2	22 ± 0.5	17 ± 1.0	5.3 ± 1.3
**Hg(II) addition after**	12	23 ± 0.7	16 ± 1.4	7.8 ± 2.1	6.7 ± 0.7	6.1 ± 0.2	1.6 ± 0.0	4.5 ± 0.1	17 ± 0.5	14 ± 1.5	3.3 ± 2.3
**6 h**	30	18 ± 2.0	12 ± 0.7	6.0 ± 2.6	12 ± 2.0	6.7 ± 0.4	1.6 ± 0.1	5.1 ± 0.3	11 ± 1.6	10 ± 0.8	0.8 ± 3.0
**(b) Aged cells in**	6.5	34 ± 5.0	26 ± 5.1	7.4 ± 10	0.3 ± 5.0	1.8 ± 0.6	0.4 ± 0.1	1.4 ± 0.5	32 ± 5.3	26 ± 5.2	6.1 ± 11
**metabolite assay,**	8	32 ± 5.9	26 ± 5.0	6.6 ± 11	1.7 ± 5.9	3.5 ± 1.8	0.8 ± 0.4	2.7 ± 1.4	29 ± 6.5	25 ± 5.4	3.9 ± 12
**Hg(II) addition after**	12	30 ± 7.5	21 ± 5.6	8.6 ± 13	0.0 ± 7.5	4.7 ± 1.7	1.2 ± 0.4	3.5 ± 1.2	25 ± 8.4	20 ± 6.0	5.1 ± 14
**6 h**	30	27 ± 4.2	16 ± 3.3	12 ± 7.5	2.7 ± 4.2	8.7 ± 0.9	3.7 ± 0.4	5.0 ± 0.5	19 ± 3.5	12 ± 3.7	6.6 ± 8.1
	0.5	21 ± 1.0	14 ± 0.0	7.2 ± 1.1	9.2 ± 1.0	0.9 ± 0.1	0.2 ± 0.0	0.7 ± 0.1	20 ± 1.2	13 ± 0.1	6.5 ± 1.2
	2	20 ± 0.3	13 ± 1.3	7.3 ± 1.7	9.7 ± 0.3	1.9 ± 0.2	0.3 ± 0.0	1.5 ± 0.2	18 ± 0.1	13 ± 1.4	5.8 ± 1.8
**(c) Metabolite**	5	17 ± 0.7	13 ± 1.2	4.6 ± 1.9	13 ± 0.7	3.6 ± 0.4	0.8 ± 0.1	2.8 ± 0.3	14 ± 0.3	12 ± 1.3	1.9 ± 2.2
**addition after 6 h in**	6.5	16 ± 2.1	11 ± 1.4	4.7 ±3.5	14 ± 2.1	3.6 ± 0.6	2.1 ± 0.4	1.5 ± 0.2	13 ± 1.5	9.3 ± 1.7	3.2 ± 3.7
**standard assay**	8	15 ± 1.8	11 ± 1.8	4.5 ± 3.6	15 ± 1.8	3.6 ± 0.6	2.1 ± 0.4	1.5 ± 0.3	11 ± 1.2	8.4 ± 2.1	3.0 ± 3.8
	12	13 ± 2.3	8.4 ± 1.0	4.7 ± 3.3	17 ± 2.3	3.8 ± 0.7	1.4 ± 0.3	2.5 ± 0.5	9.2 ± 1.6	7.0 ± 1.3	2.2 ± 3.7
	30	13 ± 2.6	7.5 ± 0.8	5.8 ± 3.4	17 ± 2.6	5.6 ± 1.0	1.8 ± 0.3	3.8 ± 0.7	7.8 ± 1.7	5.7 ± 1.1	2.1 ± 4.1

Table S8: LMM-thiol concentrations (nM) of the three additional assay systems: (a) aged cells in standard assay, (b) aged cells in metabolite assay and (c) metabolite addition after 6 h in standard assay at different time points (h) (n=3; ± standard deviation).[a]

	**Time**	**Cys**	**CysN**	**HCys**	**Glyc**	**MAC**	**Σ small LMM-RSH**	**PEN**	**NacCys**	**Σ branched LMM-RSH**	**Σ LMM-RSH**
	(h)	(nM)	(nM)	(nM)	(nM)	(nM)	(nM)	(nM)	(nM)	(nM)	(nM)
	0.5	0.8 ± 1.2	82 ± 15	1.2 ± 1.7	1.7 ± 0.8	2.5 ± 0.5	88 ± 36	-	1.0 ± 0.2	1.0 ± 0.7	89
**(a) Aged cells in**	5	1.5 ± 2.1	100 ± 11	0.2 ± 0.3	4.1 ± 5.8	0.1 ± 0.1	110 ± 45	1.2 ± 1.5	5.4 ± 1.7	6.6 ± 2.9	120
**standard assay,**	6.5	3.0 ± 1.0	130 ± 53	1.7 ± 1.2	0.2 ± 0.3	2.2 ± 0.4	130 ± 56	0.6 ± 0.6	7.8 ± 0.2	8.4 ± 5.1	140
**Hg(II) addition after**	8	3.8 ± 5.3	150 ± 22	0.1 ± 0.2	6.3 ± 7.1	0.4 ± 0.2	160 ± 67	1.2 ± 0.2	9.1 ± 4.4	10 ± 5.6	170
**6 h**	12	4.1 ± 0.5	160 ± 2.0	-	0.4 ± 0.6	-	170 ± 73	-	7.1 ± 1.8	7.1 ± 5.0	180
	30	19 ± 2.8	100 ± 25	0.2 ± 0.2	1.5 ± 0.9	-	120 ± 43	2.4 ± 0.1	8.3 ± 2.3	11 ± 4.2	130
	0.5	240 ± 180	30 ± 50	1.5 ± 1.3	1.2 ± 2.0	1.6 ± 2.7	270 ± 100	3.0 ± 1.4	6.2 ± 2.3	9.3 ± 2.2	280
**(b) Aged cells in**	5	120 ± 76	31 ± 51	-	0.8 ± 1.4	0.1 ± 0.1	150 ± 51	10 ± 4.9	8.6 ± 2.1	19 ± 1.3	170
**metabolite assay,**	6.5	50 ± 32	59 ± 99	1.0 ± 1.8	0.8 ± 1.4	1.7 ± 3.0	110 ± 29	13 ± 6.2	9.9 ± 2.9	23 ± 2.2	140
**Hg(II) addition after**	8	43 ± 31	48 ± 78	0.4 ± 0.5	0.1 ± 0.2	0.2 ± 0.3	92 ± 25	31 ± 28	7.6 ± 4.8	39 ± 17	130
**6 h**	12	41 ± 30	42 ± 69	1.0 ± 1.4	0.0 ± 0.0	-	85 ± 22	64 ± 48	11 ± 4.1	76 ± 38	160
	30	250 ± 210	23 ± 35	1.4 ± 1.1	1.7 ± 3.0	-	270 ± 110	100 ± 54	13 ± 4.0	110 ± 61	390
	0.5	0.5 ± 0.2	52 ± 12	0.2 ± 0.1	1.5 ± 2.2	2.1 ± 0.7	56 ± 22	0.1 ± 0.1	0.6 ± 0.1	0.6 ± 0.3	57
	2	0.5 ± 0.6	60 ± 8.5	0.2 ± 0.1	4.1 ± 3.1	1.2 ± 0.8	65 ± 26	-	1.4 ± 0.5	1.4 ± 1.0	67
**(c) Metabolite**	5	13 ± 4.1	81 ± 37	0.4 ± 0.5	6.3 ± 1.4	0.1 ± 0.1	100 ± 35	0.5 ± 0.7	14 ± 2.5	14 ± 9.3	120
**addition after 6 h in**	6.5	460 ± 200	120 ± 47	0.3 ± 0.1	0.2 ± 0.3	0.7 ± 0.5	580 ± 200	2.3 ± 0.3	14 ± 2.4	16 ± 8.1	600
**standard assay**	8	440 ± 240	120 ± 18	2.0 ± 2.2	2.2 ± 0.2	1.1 ± 0.3	560 ± 190	5.1 ± 0.9	20 ± 2.3	25 ± 10	590
	12	72 ± 5.8	140 ± 15	0.2 ± 0.2	1.8 ± 0.0	-	210 ± 61	66 ± 10	17 ± 1.7	84 ± 35	290
	30	39 ± 2.2	68 ± 39	0.5 ± 0.4	0.9 ± 1.3	0.1 ± 0.0	110 ± 31	120 ± 7.0	20 ± 2.6	140 ± 69	250

[a] Cells marked with “-“ when values are under detection limit

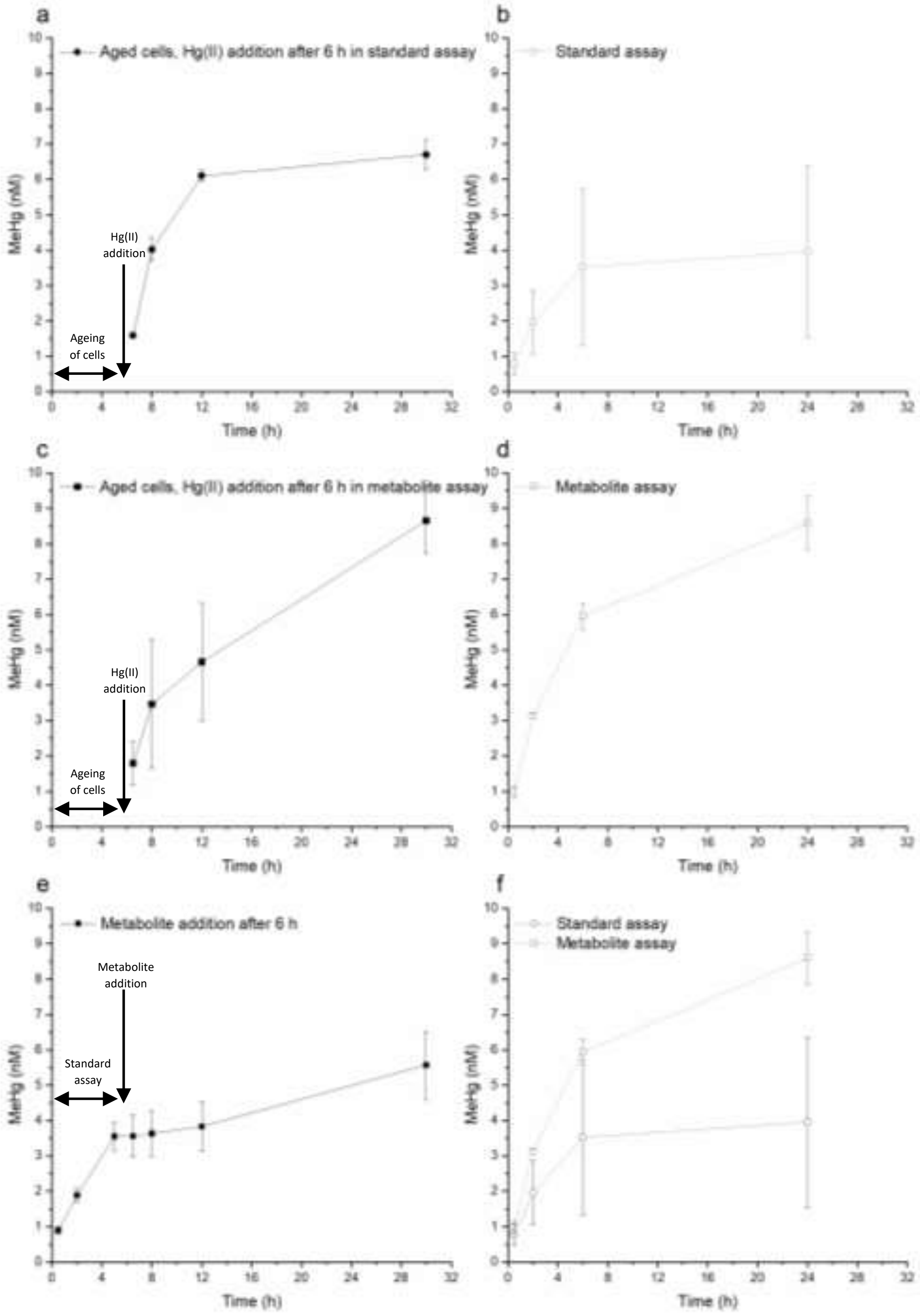

Figure S1: Methylmercury formation (nM) of *G. sulfurreducens* over time (h) in standard and metabolite assay with 30 nM Hg(II). (a) Standard assay with aged cells for 6 h Hg(II) addition occurred after 6 h (filled squares), (b) standard assay (open squares, same data as in Figure 1), (c) Metabolite assay with aged cells for 6 h and Hg(II) addition occurred after 6 h (filled circles), (d) Metabolite assay (open circles, same data as in Figure 1), (e) Standard assay for 6 h and after 6 h addition of 10% (v/v) metabolite medium (filled rectangles), (f) standard assay (open squares, same data as in Figure 1) and metabolite assay (open circles, same data as in Figure 1). In figure b, d and f the Hg(II) addition occurred at time point t = 0 h. (n=3; ± standard deviation)

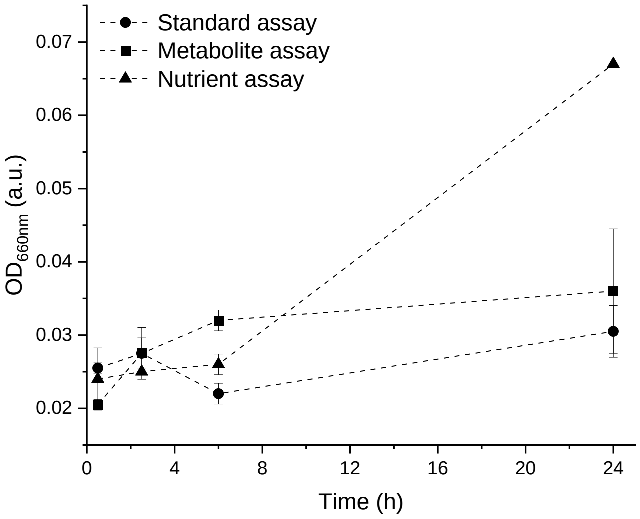

Figure S2: Cell density (OD_{660nm}) of *G. sulfurreducens* over time (h) in standard (circle), nutrient (triangle) and metabolite assay (squares) over time (h) (n=2; ± standard deviation).

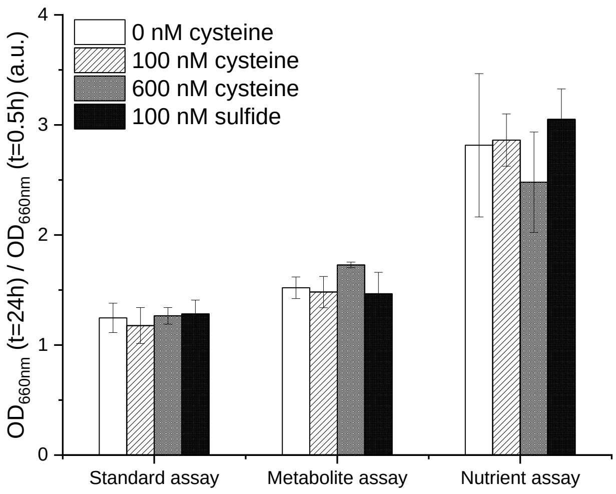

Figure S3: Cell density changes of *G. sulfurreducens* in standard, metabolite and nutrient assay with exogenous cysteine of 0, 100 and 600 nM or 100 nM sulfide illustrated by normalizing the measured OD_{660nm} at 24 h with the initial measured OD_{660nm} at 0.5 h (OD_{660nm}(t=24h)/OD_{660nm}(t=0.5h)); (n=3; ± standard deviation).

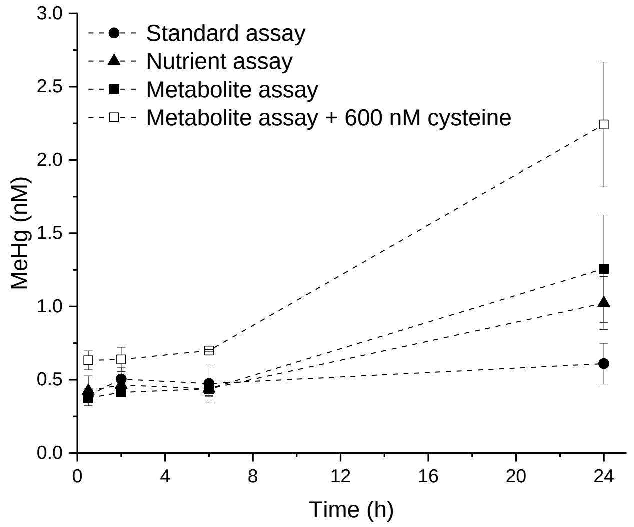

Figure S4: Methylmercury formation (nM) under sulfidic condition by *G. sulfurreducens* in standard (circle filled), nutrient (triangle filled), metabolite (square filled) and metabolite + 600 nM cysteine (square open symbol) over time (h) with exogenous sulfide of 100 nM (n=3; ± standard deviation).

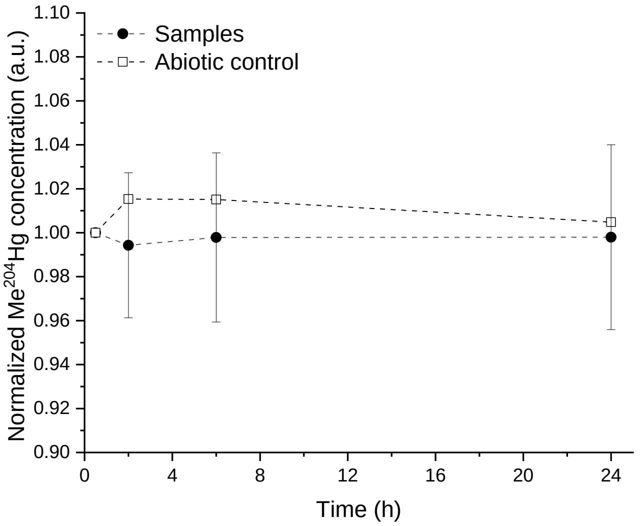

Figure S5: Demethylation of MeHg in biotic (with *G. sulfurreducens*; circles filled) and abiotic (without *G. sulfurreducens*; triangles open) assays by tracing $Me^{204}Hg$ changes over time (h) by normalizing the measured $Me^{204}Hg$ concentrations at the different time points to the initial measured $Me^{204}Hg$ concentrations at t = 0.5 h (n=38 for biotic samples; ± standard deviation).

Quality control and Hg recovery

Isotope dilution analysis

The determination of total Hg and MeHg was performed with isotope dilution analysis (IDA) by ICP-MS. Herby an isotopic enriched Hg standard, in this case ^{200}Hg (ORNL batch 185091) or $Me^{200}Hg$ (synthesized in-house according to Snell et al. from $^{200}Hg(II)$ ORNL batch 185091),[17] respectively, was added as an internal standard to the sample.[18] Isotope dilution technique is a definitive and highly reliable method due to its precision and accuracy, since losses (including degradation) of the analyte in the sample are corrected for provided that fundamental criteria of IDA are fulfilled. Of major importance to acquire reliable results is to achieve an isotope equilibration in the sample and to use a traceable primary standard.[19] The concentrations of the prepared ^{200}Hg and $Me^{200}Hg$ isotope standards were determined by reversed isotope dilution (RIDA) in connection to their use for IDA quantification using the same analytical methods as for total Hg and MeHg determinations. The concentrations were determined using a TraceCERT®, 1000 mg/L ± 4 mg/L Hg in nitric acid stock solution and a 1.44 mM MeHg in 0.1 M HCl stock solution prepared gravimetrically from MeHgCl (PESTANAL®, analytical standard, ≥98.0 %), respectively as primary standards. The uncertainty in the ^{200}Hg and $Me^{200}Hg$ isotope standard concentrations from the RIDA determinations were 0.9 and 3.5 %, respectively.

Control samples

Two different control samples were processed and analyzed in triplicates (n=3) to exclude (I) abiotic MeHg formation and (II) Hg contamination during sample processing. An abiotic control sample (control I) with 30 nM Hg(II) in standard assays without cysteine and in the absence of *G. sulfurreducens* cells was incubatedfor 0.5 h. A biotic control sample (control II) without Hg(II) and

in the presence of *G. sulfurreducens* cells was incubated for 0.5 h. The recovery of total and dissolved Hg concentration for control I was 23.6 ± 1.2 and 22.3 ± 1.1 nM and the measured MeHg concentration was 0.18 ± 0.02 nM. The loss of Hg in the abiotic control I thus corresponded to ~22%. Control II showed total and dissolved Hg concentration of 0.75 ± 0.25 and 0.70 ± 0.13 nM and the measured MeHg concentration was 0.22 ± 0.06 nM. These concentrations were negligible compared to the experimental assays with added Hg(II) and cells.

Moreover, a control sample in the absence of *G. sulfurreducens* cells in metabolite assays without cysteine addition was performed during the whole incubation time of 24 h. The concentration of total Hg and dissolved Hg decreased by ~28% between Hg(II) addition and 0.5 h and by an additional ~15 % between 0.5 and 24 h The measured total MeHg remained low and varied between 0.5 – 0.8 nM during the whole incubation time and is in agreement with control I and II.

<u>Recovery of Hg</u>

The recovery of total Hg in 100 mL glass serum vials closed with Teflon stopper in the presence and absence of *G. sulfurreducens* cells is shown in Figure S6. The Hg recovery experiment was performed by adding 37 nM Hg(II) as $HgCl_2$ to each assay vials and equilibrating for 1 h. In the biotic samples, inoculation with cells was done after 1 h equilibration and the assays were then incubated for 6 h. Both abiotic and biotic assay vials were then purged for 15 min with N_2, and a sample aliquot of the remaining solution was collected and digested with BrCl for total Hg analysis. This sample is referred to as "Hg(suspended, non-purgeable)" and represents the sum of dissolved and cell associated Hg in the assays. Subsequently, BrCl was added to the assay glass vessel to digest the entire non-purgeable assay sample. This sample is referred to as "Hg(assay vial, non-purgeable)" and represents to sum of the "Hg(suspended, non-purgeable)" and vessel adsorbed Hg.. Both samples were processed and analyzed as described in the material and method section.

Based on these measurements, the amount of vial adsorbed Hg was calculated as:

Hg(vial adsorbed) = Hg(assay vial, non-purgeable) – Hg(suspended, non-purgeable)

The amount of purgeable Hg was calculated as the difference between the added Hg, referred to as "Hg(added)" and the determined Hg(assay vial, non-purgeable) amount:

Hg(purgeable) = Hg(added) – Hg(assay vial, non-purgeable)

It is assumed that the Hg(purgeable) fraction corresponds to the amount of Hg(II) reduced to Hg(0) in the assays. We here define Hg losses as the sum of the Hg(vessel adsorbed) and Hg(purgeable) fractions. All assay buffer showed losses of 15 – 25 % of the added Hg(II) in samples purged after 1 h equilibration without cells (Figure S6a). In samples purged after 6 h incubation in the presence of cells, the Hg losses were 10 – 30 % (Figure S6b). The majority of losses thus occurred during the 1 h equilibration of Hg without cells. The partitioning between vial adsorbed and suspended, non-purgeable Hg did however change during Hg equilibration and cell incubation. A small fraction of Hg was adsorbed to the glass vial surface after 1 h equilibration with 4 - 10 % of the added Hg, whereas after 6 h incubation with cells the vial adsorbed Hg fraction had increased. The highest Hg adsorption was observed for standard assay with ~30% of the added Hg. Metabolite and nutrient assays with cells showed an intermediate increase of the adsorbed Hg fraction with ~15% of the added Hg. Consequently, the recovery of added Hg after 6 h was ~40% in the standard assay and ~70% in metabolite and nutrient assays. The suspended, non-purgeable Hg decreased over time and in the presence of cells (Figure S6, Table S2). Losses of Hg were higher under conditions with lower ligand concentrations (Table S1 and S3). Overall, in the presence of cells and after 6 h incubation, loses of Hg by vial adsorption and reduction were of similar magnitude.

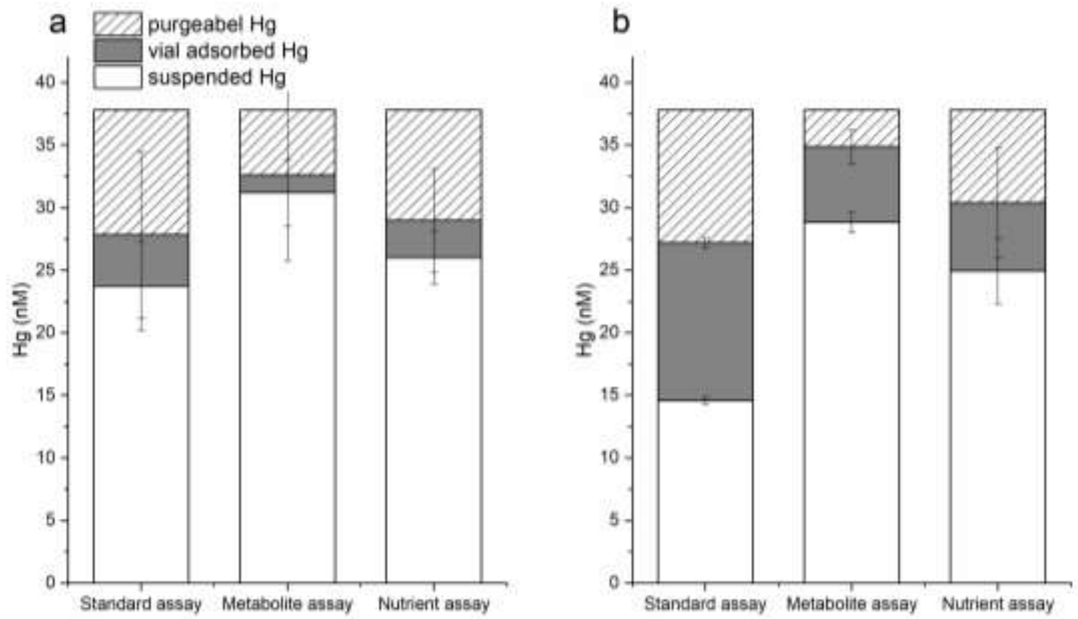

Figure S6: Recovery of 37 nM Hg(II) in 100 mL anoxic closed glass serum vials in standard, metabolite and nutrient assay buffer. (a) Abiotic control samples after 1 h equilibration with Hg(II) and (b) *G. sulfurreducens* cells were added after 1 h equilibration with Hg(II) and cells were incubated for 6 h. (n = 2)

REFERENCES

1. Schaefer, J. K.; Rocks, S. S.; Zheng, W.; Liang, L. Y.; Gu, B. H.; Morel, F. M. M., Active transport, substrate specificity, and methylation of Hg(II) in anaerobic bacteria. *Proc. Natl. Acad. Sci. U. S. A.* **2011,** *108*, (21), 8714-8719.
2. Schaefer, J. K.; Szczuka, A.; Morel, F. M. M., Effect of Divalent Metals on Hg(II) Uptake and Methylation by Bacteria. *Environmental Science & Technology* **2014,** *48*, (5), 3007-3013.
3. Adediran, G. A.; Liem-Nguyen, V.; Song, Y.; Schaefer, J. K.; Skyllberg, U.; Bjorn, E., Microbial Biosynthesis of Thiol Compounds: Implications for Speciation, Cellular Uptake, and Methylation of Hg(II). *Environmental Science & Technology* **2019,** *53*, (14), 8187-8196.
4. Lin, H.; Lu, X.; Liang, L.; Gu, B., Thiol-Facilitated Cell Export and Desorption of Methylmercury by Anaerobic Bacteria. *Environmental Science & Technology Letters* **2015,** *2*, (10), 292-296.
5. Gutensohn, M.; Schaefer, J. K.; Maas, T. J.; Skyllberg, U.; Björn, E., Metabolic turnover of cysteine-related thiol compounds at environmentally relevant concentrations by Geobacter sulfurreducens. *Frontiers in Microbiology* **2023,** *13*.
6. Karlsson, M.; Lindgren, J. http://www.winsgw.se/WinSGWeng.htm. http://www.winsgw.se/WinSGWeng.htm (June 06),
7. Smith, R. M., NIST Critically Selected Stability Constant of metal complexes database. *Version 4* **1997**.
8. Milero, F. J., The thermodynamics and kinetics of the hydrogen sulfide system in natural waters. *Mar. Chem.* **1986,** *18*, (2-4), 121-147.
9. Liem-Nguyen, V.; Skyllberg, U.; Nam, K.; Bjorn, E., Thermodynamic stability of mercury(II) complexes formed with environmentally relevant low-molecular-mass thiols studied by competing ligand exchange and density functional theory. *Environ. Chem.* **2017,** *14*, (4), 243-253.
10. Walmsley, A. R.; Shaw, J. G.; Kelly, D. J., Perturbation of the equilibrium between open and closed conformations of the periplasmic C4-dicarboxylate binding protein from Rhodobacter capsulatus. *Biochemistry* **1992,** *31*, (45), 11175-81.
11. Dyrssen, D.; Wedborg, M., The sulphur-mercury(II) system in natural waters. *Water, Air & Soil Pollution* **1991,** *56*, (1), 507-519.
12. Schwarzenbach, G.; Widmer, M., Die Löslichkeit von Metallsulfiden I. Schwarzes Quecksilbersulfid. *Helvetica Chimica Acta* **1963,** *46*, (7), 2613-2628.
13. Ravichandran, M., Interactions between mercury and dissolved organic matter--a review. *Chemosphere* **2004,** *55*, (3), 319-31.
14. Cardiano, P.; Falcone, G.; Foti, C.; Sammartano, S., Sequestration of Hg2+by Some Biologically Important Thiols. *Journal of Chemical & Engineering Data* **2011,** *56*, (12), 4741-4750.
15. Jalilehvand, F.; Leung, B. O.; Izadifard, M.; Damian, E., Mercury(II) Cysteine Complexes in Alkaline Aqueous Solution. *Inorg. Chem.* **2006,** *45*, (1), 66-73.
16. Drott, A.; Bjorn, E.; Bouchet, S.; Skyllberg, U., Refining thermodynamic constants for mercury(II)-sulfides in equilibrium with metacinnabar at sub-micromolar aqueous sulfide concentrations. *Environmental Science & Technology* **2013,** *47*, (9), 4197-203.
17. Snell, J. P.; Stewart, I. I.; Sturgeon, R. E.; Frech, W., Species specific isotope dilution calibration for determination of mercury species by gas chromatography coupled to inductively coupled plasma- or furnace atomisation plasma ionisation-mass spectrometry. *Journal of Analytical Atomic Spectrometry* **2000,** *15*, (12), 1540-1545.
18. Björn, E.; Larsson, T.; Lambertsson, L.; Skyllberg, U.; Frech, W., Recent advances in mercury speciation analysis with focus on spectrometric methods and enriched stable isotope applications. *Ambio* **2007,** *36*, (6), 443-51.
19. Heumann, K. G., Book Review: Guidelines for Achieving High Accuracy in Isotope Dilution Mass Spectrometry (IDMS) Edited by Mike Sargent, Chris Harrington and Rita Harte. *Angewandte Chemie International Edition* **2003,** *42*, (14), 1564-1565.
